# Type II Secretion-Dependent Aminopeptidase LapA and Acyltransferase PlaC Are Redundant for Nutrient Acquisition during *Legionella pneumophila* Intracellular Infection of Amoebas

**DOI:** 10.1128/mBio.00528-18

**Published:** 2018-04-17

**Authors:** Richard C. White, Felizza F. Gunderson, Jessica Y. Tyson, Katherine H. Richardson, Theo J. Portlock, James A. Garnett, Nicholas P. Cianciotto

**Affiliations:** a Department of Microbiology and Immunology, Northwestern University Medical School, Chicago, Illinois, USA; b Department of Chemistry and Biochemistry, Queen Mary University of London, London, United Kingdom; University of Michigan-Ann Arbor

**Keywords:** *Acanthamoeba*, acyltransferase, aminopeptidase, *Legionella pneumophila*, Legionnaires' disease, protease, type II secretion, intracellular infection

## Abstract

Legionella pneumophila genes encoding LapA, LapB, and PlaC were identified as the most highly upregulated type II secretion (T2S) genes during infection of Acanthamoeba castellanii, although these genes had been considered dispensable on the basis of the behavior of mutants lacking either *lapA* and *lapB* or *plaC*. A *plaC* mutant showed even higher levels of *lapA* and *lapB* transcripts, and a *lapA lapB* mutant showed heightening of *plaC* mRNA levels, suggesting that the role of the LapA/B aminopeptidase is compensatory with respect to that of the PlaC acyltransferase. Hence, we made double mutants and found that *lapA plaC* mutants have an ~50-fold defect during infection of A. castellanii. These data revealed, for the first time, the importance of LapA in any sort of infection; thus, we purified LapA and defined its crystal structure, activation by another T2S-dependent protease (ProA), and broad substrate specificity. When the amoebal infection medium was supplemented with amino acids, the defect of the *lapA plaC* mutant was reversed, implying that LapA generates amino acids for nutrition. Since the LapA and PlaC data did not fully explain the role of T2S in infection, we identified, via proteomic analysis, a novel secreted protein (NttD) that promotes infection of A. castellanii. A *lapA plaC nttD* mutant displayed an even greater (100-fold) defect, demonstrating that the LapA, PlaC, and NttD data explain, to a significant degree, the importance of T2S. LapA-, PlaC-, and NttD-like proteins had distinct distribution patterns within and outside the *Legionella* genus. LapA was notable for having as its closest homologue an A. castellanii protein.

## IMPORTANCE

Transmission of L. pneumophila to humans is facilitated by its ability to grow in *Acanthamoeba* species. We previously documented that type II secretion (T2S) promotes L. pneumophila infection of A. castellanii. Utilizing transcriptional analysis and proteomics, double and triple mutants, and crystal structures, we defined three secreted substrates/effectors that largely clarify the role of T2S during infection of A. castellanii. Particularly interesting are the unique functional overlap between an acyltransferase (PlaC) and aminopeptidase (LapA), the broad substrate specificity and eukaryotic-protein-like character of LapA, and the novelty of NttD. Linking LapA to amino acid acquisition, we defined, for the first time, the importance of secreted aminopeptidases in intracellular infection. Bioinformatic investigation, not previously applied to T2S, revealed that effectors originate from diverse sources and distribute within the *Legionella* genus in unique ways. The results of this study represent a major advance in understanding *Legionella* ecology and pathogenesis, bacterial secretion, and the evolution of intracellular parasitism.

## INTRODUCTION

One of 62 species in *Legionella* (1), Legionella pneumophila is an inhabitant of water systems and the main agent of Legionnaires’ disease, a pneumonia of increasing prevalence (2, 3). Disease largely results from inhalation of contaminated droplets from aerosolizing devices (4). In aquatic habitats, the Gram-negative bacterium L. pneumophila flourishes as an intracellular parasite of amoebas, especially species of *Acanthamoeba*, *Vermamoeba*, and *Naegleria* (5, 6). Within lungs, L. pneumophila grows in macrophages, residing, as it does in amoebas, in a membrane-bound compartment, the *Legionella*-containing vacuole (LCV) (7, 8). Critical to infection is a type IV secretion system (T4SS), which translocates myriad effectors from the LCV into the host cytoplasm (9–11).

Type II secretion (T2S) is another major factor in L. pneumophila pathogenesis (12, 13). The T2S system (T2SS) promotes infection of the lungs, enhancing *Legionella* growth in macrophages, dampening the host cytokine response, and releasing damaging enzymes (14–16). While the T2SS does not impact entry into macrophages or evasion of lysosomes, it does promote replication in the LCV at 4 to 12 h postentry (17). T2S is also critical for L. pneumophila survival in water and for infection of Acanthamoeba castellanii, Naegleria lovaniensis, and Vermamoeba vermiformis (18–24). T2S also potentiates L. pneumophila biofilm formation and sliding motility (25, 26). During T2S, protein substrates first move across the inner membrane via the Sec or Tat pathway; once in the periplasm, they are recognized by the T2SS apparatus, which uses a pseudopilus to drive the proteins through an outer membrane channel (13, 27). On the basis of proteomic and enzymatic analysis of supernatants, we discerned that L. pneumophila T2S elaborates at least 27 proteins, including nearly 20 enzymes and novel proteins of unknown activity (13, 28, 29). T2S-dependent proteins known to promote infection of key amoebal hosts include the novel NttA protein, which facilitates infection of A. castellanii; the novel NttC protein, which fosters infection of V. vermiformis; and acyltransferase PlaC, metalloprotease ProA, and RNase SrnA, which enhance infection of V. vermiformis and N. lovaniensis (23, 24, 30, 31). Overall, L. pneumophila T2S is one of the best-characterized T2SSs, in terms of its output and functions (13). Yet there are gaps in knowledge; e.g., the exoproteins thus far linked to infection do not fully explain the impact of T2SS on L. pneumophila ecology. On the basis of a form of transcriptional analysis and proteomics, we have now used genetic, biochemical, bioinformatic, and structural biology methods to define a eukaryotic-protein-like aminopeptidase and a novel secreted protein that provide unique insight into the evolution of T2S and parasitism.

## RESULTS

### Transcriptional analysis identified T2SS-dependent LapA, LapB, and PlaC as hyperexpressed in a compensatory manner during infection of A. castellanii.

**FIG 1:**
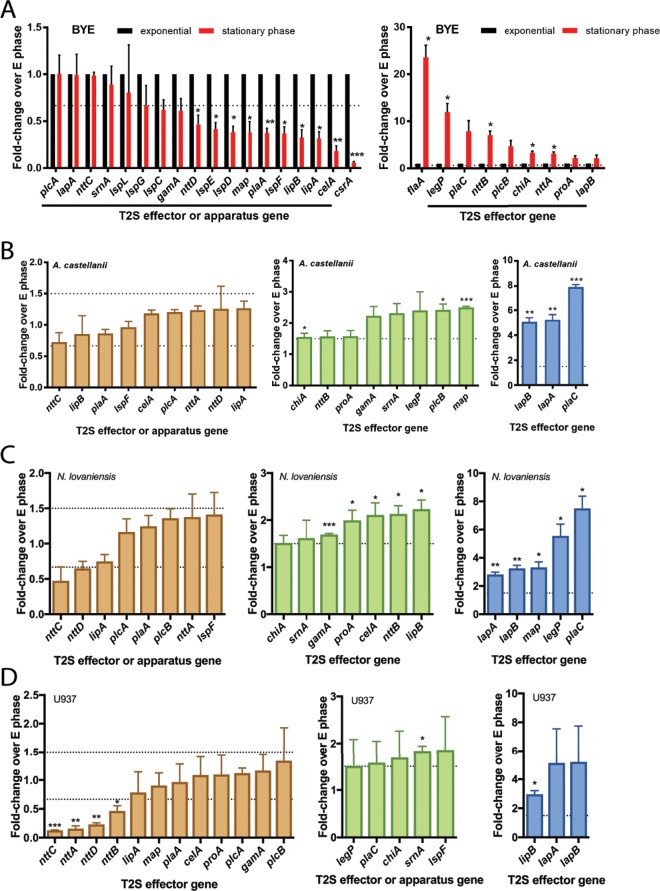
Relative expression levels of genes encoding type II-secreted proteins (effectors) in wild-type L. pneumophila grown in broth or within amoebas and human macrophages. (A) Gene expression within wild-type 130b bacteria grown in BYE broth to the exponential phase (E phase) or stationary phase. qRT-PCR data are presented as means with standard errors of results from three independently grown cultures, with expression in the stationary phase normalized to expression in the E phase in broth. (B to D) Gene expression within 130b bacteria obtained from infected A. castellanii (B) or N. lovaniensis (C) at 44 h postinoculation or from infected human U937 macrophages (D) at 12 h postinoculation. qRT-PCR data are presented as means with standard errors of results from three independent infections (*n* = 3 each), with the intracellular expression normalized to the E phase in broth. Dashed lines indicate the customary 1.5-fold up- and/or downregulation threshold of biological interest. Asterisks indicate significant differences with >1.5-fold change relative to the E phase (*, *P* < 0.05; **, *P* < 0.01; ***, *P* < 0.001 [one-sample *t* test]).

As a new means of uncovering important secreted proteins, we identified T2S substrates whose level of gene transcription is upregulated during infection of amoebas. We harvested wild-type strain 130b bacteria from both infected A. castellanii and exponential-phase buffered yeast extract (BYE) cultures, isolated RNA, and used quantitative reverse transcriptase PCR (qRT-PCR) to compare gene expression levels in intracellular versus extracellular bacteria. Since past studies identified some infectivity factors as being upregulated during the stationary phase (32), we also analyzed expression in stationary-phase cultures. Whereas five of the secreted-protein genes showed no change in expression in comparisons of log-phase versus stationary-phase bacteria, six others, along with the non-T2S *csrA* gene (33), were downregulated during the stationary phase (Fig. 1A, left). In contrast, eight other substrate genes, along with non-T2S *flaA* (32), had or trended toward increased expression in stationary phase (Fig. 1A, right). We also saw that the expression levels of genes encoding the T2SS apparatus were unchanged or downregulated in the stationary phase (Fig. 1A, left). Analyzing intracellularly grown bacteria, we found that 8/19 secreted-protein genes, along with *lspF*, which is representative of the T2SS apparatus, were expressed at a level that was equal to or slightly less than that seen in broth-grown bacteria (Fig. 1B, left). Eight other substrate genes were upregulated ~2-fold in intracellular bacteria (Fig. 1B, middle). Most notably, *lapA*, *lapB*, and *plaC* displayed 5- to 8-fold-higher levels in bacteria from within amoebas (Fig. 1B, right), suggesting that aminopeptidases LapA and LapB, which share 42% identity (30), and acyltransferase PlaC (34) are very important during intracellular growth.

**FIG 2:**
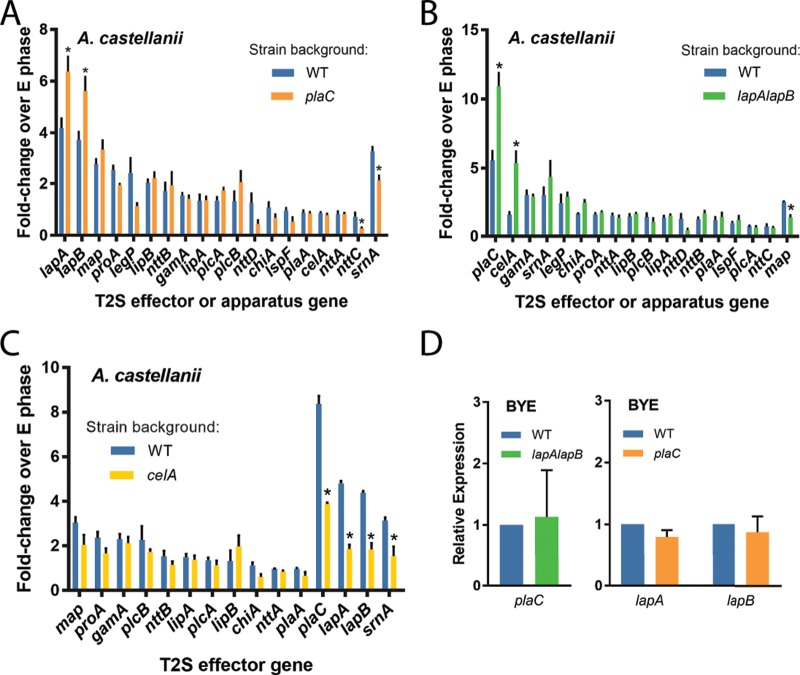
Relative expression levels of genes encoding type II-secreted protein effectors in *plaC* mutant, *lapA lapB* mutant, and *celA* mutant bacteria grown in A. castellanii or in broth. (A to C) Gene expression within wild-type strain 130b (WT) versus *plaC* mutant NU420 (A), WT versus *lapA lapB* mutant NU324 (B), or WT versus *celA* mutant NU353 (C) obtained from infected A. castellanii at 44 h postinoculation. qRT-PCR data are represented as means with standard errors of results from three independent infections (*n* = 3 each), with expression normalized to the exponential (E) phase in broth. Asterisks indicate significant differences with >1.5-fold change in expression relative to the WT (*, *P* < 0.05 [Student’s *t* test]). (D) Gene expression in WT and *lapA lapB* mutant NU324 (left) or *plaC* mutant NU420 (right) during E phase in broth. Data are presented as means with standard errors of results from three independent cultures (*n* = 3), with expression normalized to the WT results.

When we examined, in a similar way, gene expression in a *plaC* mutant (NU420) (23), we detected an even higher level of expression of *lapA* and *lapB*, while the expression levels of other T2S genes were unchanged or slightly decreased (Fig. 2A). In contrast, when we assayed transcript levels in a *lapA lapB* mutant (NU324) (30), there was a further heightening in levels of *plaC* mRNA (Fig. 2B). We also detected an increase in *celA* transcription in the *lapA lapB* mutant (Fig. 2B), but, when we examined expression in a *celA* mutant (NU353) (29), there was no compensatory increase in expression of *lapA* or *lapB* or of any other genes (Fig. 2C). These data suggested that the intracellular role(s) of LapA/LapB might be functionally compensatory with respect to that of PlaC, such that when the aminopeptidases are absent there is an increase in acyltransferase levels and vice versa. When the *plaC* mutant and *lapA lapB* mutant were grown in broth, no compensatory changes in expression were seen (Fig. 2D), implying that the functional redundancy between PlaC and LapA/LapB may be specific to intracellular growth.

### Double mutants reveal key roles for LapA and PlaC in infection of A. castellanii.

**FIG 3:**
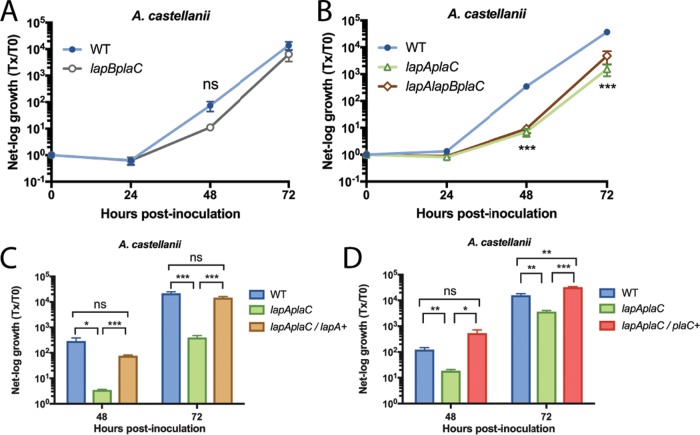
Intracellular infection of A. castellanii by L. pneumophila wild-type, *lapB plaC* mutant, *lapA plaC* mutant, and *lapA lapB plaC* mutant strains. (A and B) A. castellanii amoebas were infected with either wild-type strain 130b (WT) and *lapB plaC* mutant NU433 (A) or WT, *lapA plaC* mutant NU435, and *lapA lapB plaC* mutant NU436 (B). At the indicated times, net bacterial growth was determined by plating for CFU counts. Data are presented as means with standard errors of results from three independent experiments (*n* = 3 each). Tx/T0, determination time versus time 0. (C and D) Amoebas were infected with WT, *lapA plaC* mutant NU435, and complemented mutant NU435 containing plasmid-carried *lapA* (C) or plasmid-carried *plaC* (D). Bacterial growth data are presented as means with standard deviations of results from four infected wells and are representative of three independent experiments. Asterisks indicate significant differences in CFU recovery between a mutant and the WT or its complemented derivative (*, *P* < 0.05; **, *P* < 0.01; ***, *P* < 0.001 [Student’s *t* test]). ns, not significant.

We previously observed that neither *plaC* mutant NU420 nor *lapA lapB* mutant NU324 is impaired for infection of A. castellanii (23, 30). Given our transcriptional data, we posited that a *lapA plaC* mutant, a *lapB plaC* mutant, and/or a *lapA lapB plaC* mutant might be impaired for infection due to the combined loss of two or more functionally redundant genes. Thus, we made double and triple mutants, and after observing their capacity to grow typically in BYE medium and survive normally in conditioned amoebal medium (see Fig. S1A and B [left panels] in the supplemental material), we tested them for infection of A. castellanii. Whereas the *lapB plaC* mutant (NU433) grew similarly to the wild type (Fig. 3A), the *lapA plaC* mutant (NU435) displayed an ~50-fold defect in infection (Fig. 3B). The *lapA lapB plaC* mutant (NU436) was no more defective than the *lapA plaC* mutant (Fig. 3B). Since *lapA* and *plaC* are monocistronic (Fig. S2A and B), the mutant defect was likely due to loss of *lapA* and *plaC* rather than polarity on downstream genes. When *lapA* was reintroduced into the *lapA plaC* mutant, there was restoration of infection (Fig. 3C), confirming the role of LapA. Similarly, the *lapA plaC* mutant was complemented by reintroduction of *plaC* (Fig. 3D), affirming the role of PlaC. Thus, we extended our transcriptional analysis of intracellular legionellae to include N. lovaniensis (Fig. 1C). Again, we saw that *lapA*, *lapB*, and *plaC* were upregulated (Fig. 1C, right). *legP* and *map* were also notably upregulated (Fig. 1C, right). Previously, we determined that the *lapA lapB* mutant is not impaired for infection of N. lovaniensis, whereas the *plaC* mutant has an ~10-fold defect (23). Given the transcription data, we compared the *lapA lapB plaC* mutant and *plaC* mutant for infection of N. lovaniensis, to possibly uncover another role for LapA (or LapB). However, the triple mutant was no more impaired than the single mutant (Fig. S3A). A like result was attained when we tested mutants in V. vermiformis (Fig. S3B). When analysis was expanded to include infected macrophages (Fig. 1D), *lapA* and *lapB* appeared to be among the most highly upregulated genes (right), whereas the change seen with *plaC* was modest (middle). Since the *lapA lapB* mutant and *plaC* mutant are not impaired in U937 cells (30, 34), we compared the *lapA lapB plaC* mutant to the wild type with respect to infection of macrophages. However, no new mutant phenotype was detected (Fig. S3C). Thus, though *lapA* and *plaC* expression was upregulated upon *Legionella* growth in two hosts, the compensatory link between LapA and PlaC was detected only in A. castellanii. Our mutant analysis, which was based on transcript data, uncovered a role for PlaC in infection of A. castellanii, expanding the idea of the protein’s importance beyond its role in infection of V. vermiformis and N. lovaniensis (23). A glycerophospholipid-cholesterol and -ergosterol acyl-transferase that has phospholipase A and lysophospholipase A activities, PlaC has been well studied biochemically (34, 35). More importantly, our transcriptional and mutational analyses revealed, for the first time, a role for the LapA aminopeptidase in an intracellular infection event; thus, we focused our next efforts on defining the biochemical, structural, and phylogenetic traits of LapA.

10.1128/mBio.00528-18.1 FIG S1  Extracellular replication and survival of the L. pneumophila wild-type strain, an *nttD* mutant, a *lapA plaC* mutant, and a *lapA lapB plaC* mutant in conditioned amoebal medium. (A) Wild-type strain 130b (WT), *lapA plaC* mutant NU435 and *lapA lapB plaC* mutant NU436 (left), and the WT strain and *nttD* mutant NU431 (right) were inoculated into BYE broth, and then, at various times postinoculation, bacterial growth was monitored spectrophotometrically. The data points represent means and standard deviations of results from duplicate cultures, and the results presented are representative of two independent experiments. (B) A. castellanii amoebas were seeded in-well, and then a 0.4-µm transwell insertion was added followed by bacterial suspensions to prevent contact between the WT strain, *lapA plaC* mutant NU435, and *lapA lapB plaC* mutant NU436 (left) or between the WT strain and *nttD* mutant NU431 (right) or with the host cells. At the indicated times, CFU counts were recorded from within the upper chamber. Data are presented as means and standard deviations of results from duplicate wells and are representative of two independent experiments. Download FIG S1, EPS file, 1.3 MB. Copyright © 2018 White et al. 2018 White et al. This content is distributed under the terms of the Creative Commons Attribution 4.0 International license.

10.1128/mBio.00528-18.2 FIG S2  L. pneumophila loci encoding type II-secreted proteins. The chromosomal regions of strain 130b containing *lapA* (A), *plaC* (B), and *nttD* (C) are depicted. The horizontal arrows denote the locations, orientations, and relative sizes of the genes. The “lpw” numbers indicated within the arrows are ORF designations used in the database. Gene names and annotations are indicated below the arrows. Sizes of the genes are given (in base pairs) above the arrows, and sizes of intergenic regions are noted between the arrows. (A) The genes adjacent but transcriptionally unlinked to *lapA* encode type IV secretion effectors (N. Shohdy, J. A. Efe, S. D. Emr, and H. A. Shuman, Proc Natl Acad Sci U S A **102:**4866–4871, 2005; E. Portier, H. Zheng, T. Sahr, D. M. Burnside, C. Mallama, C. Buchrieser, N. P. Cianciotto, and Y. Héchard, Environ Microbiol **17:**1338–1350, 2015; D. T. Isaac, R. K. Laguna, N. Valtz, and R. R. Isberg, Proc Natl Acad Sci U S A **112:**E5208–E5217, 2015). (B) The gene upstream of *plaC* is predicted to encode part of an ABC transporter, whereas the downstream gene is likely involved in glucosamine-6-phosphate metabolism (A. Gaballa and J. D. Helmann, J Bacteriol **180:**5815–5821, 1998; R. A. Tinsley, J. R. Furchak, and N. G. Walter, RNA **13:**468–477, 2007). (C) The gene upstream of *nttD* is not annotated, although tertiary structure predictions suggest that its protein product is a lipase (L. A. Kelley, S. Mezulis, C. M. Yates, M. N. Wass, and M. J. Sternberg, Nat Protoc **10:**845–858, 2015). This protein may also be a substrate for the T2S based on the detection of the protein in wild-type supernatants and the presence of a signal peptide at the protein’s N terminus (28). The two genes downstream of *nttD* are predicted to be involved in DNA rearrangement and repair (M. M. Slupska, J. H. Chiang, W. M. Luther, J. L. Stewart, L. Amii, A. Conrad, and J. H. Miller, Genes Cells **5:**425–437, 2000; C. Darrigo, E. Guillemet, R. Dervyn, and N. Ramarao, PLoS One **11:**e0163321, 2016). Download FIG S2, EPS file, 2.3 MB. Copyright © 2018 White et al. 2018 White et al. This content is distributed under the terms of the Creative Commons Attribution 4.0 International license.

10.1128/mBio.00528-18.3 FIG S3  Intracellular infection of N. lovaniensis, V. vermiformis, and human macrophages by the L. pneumophila wild-type, *plaC* mutant, or *lapA lapB plaC* mutant strain. N. lovaniensis amoebas (A), V. vermiformis amoebas (B), or U937 macrophages (C) were infected with wild-type strain 130b (WT) or *plaC* mutant NU420 (panels A and B only) or with *lapA lapB plaC* mutant NU436, and at 0, 24, 48, and 72 h postinoculation, CFU levels within the infected wells were determined. Bacterial growth resulting from intracellular infection is expressed as the ratio of the CFU count at *t* = 24, 48, or 72 h to the CFU count at *t* = 0. Data are presented as means and standard errors of results from three independent experiments (*n* = 3). Asterisks indicate significant differences in levels of CFU recovery between the WT strain and all mutants (*, *P* < 0.05 [Student’s *t* test]). Download FIG S3, EPS file, 0.7 MB. Copyright © 2018 White et al. 2018 White et al. This content is distributed under the terms of the Creative Commons Attribution 4.0 International license.

### LapA is a protease-activated, broad-spectrum aminopeptidase.

**FIG 4:**
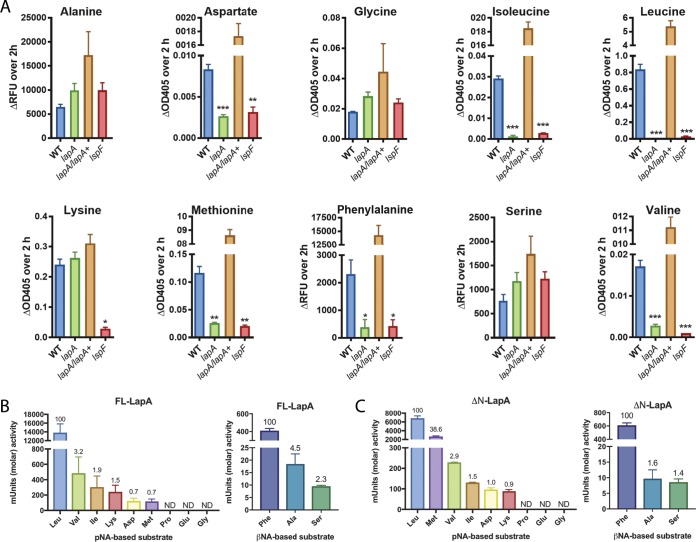
Aminopeptidase activities from L. pneumophila culture supernatants and purified recombinant LapA. Filter-sterilized supernatants from late-exponential-phase broth cultures of wild-type 130b (WT), *lapA* mutant NU320 (*lapA*), complemented *lapA* mutant NU320 containing plasmid-carried *lapA* (*lapA*/*lapA*+), and *lspF* mutant NU275 (*lspF*) were assayed for activity directed toward aminopeptidase substrates, with the N-terminal amino acid (AA) residue given. (A) Changes in background-adjusted absorbance at OD_405_ for the AA–*p*-nitroanilide substrates or fluorescence (expressed in relative fluorescence units [RFU]) for the AA–β-naphthylamide substrates were monitored kinetically over 2 h. Data are presented as means with standard errors of results from three independent experiments (*n* = 3 each). Asterisks indicate significant reductions in activity of a mutant relative to the WT (*, *P* < 0.05; **, *P* < 0.01; ***, *P* < 0.001 [Student’s *t* test]). (B and C) Strains with purified, full-length LapA (FL-LapA) (B) or LapA missing its N-terminal PA domain (ΔN-LapA) (C) were incubated for 2 h at 37°C with various AA–*p*-nitroanilide substrates (left panels) or AA–β-naphthylamide substrates (right panels), and the levels of molar activity were determined (*y* axes). The relative activities against the various substrates are displayed above each bar, with the level of activity directed toward Leu-*p*NA (B and C, left) or toward Phe-βNA (B and C, right) arbitrarily set to 100. ND, not detected. Data are presented as means with standard errors of results from two independent experiments.

Past work found that LapA is active against leucine, phenylalanine, and tyrosine aminopeptides, based on the ability of wild-type supernatants but not *lapA* mutant supernatants to cleave those substrates (30). LapA is annotated as a leucine aminopeptidase, and the Merops database assigns LapA as a member of the M28 family of peptidases (subfamily E) (28, 30, 36). We more completely defined the specificity of LapA by testing wild-type, *lspF* mutant, and *lapA* mutant supernatants against additional aminopeptides. Wild-type samples had activity against alanine, aspartate, glycine, isoleucine, lysine, methionine, serine, and valine aminopeptides, in addition to leucine and phenylalanine aminopeptides (Fig. 4A). They had no activity against proline- or glutamate-containing substrates. The *lspF* mutant lost activity against aspartate, isoleucine, leucine, lysine, methionine, phenylalanine, and valine substrates but showed normal activity against alanine, glycine, and serine aminopeptides (Fig. 4A). Thus, 7/10 of the activities are mediated by T2S. The *lapA* mutant lacked activity against aspartate, isoleucine, methionine, and valine aminopeptides and cleaved alanine, glycine, lysine, and serine substrates (Fig. 4A). As described before (30), the mutant lacked activity against leucine and phenylalanine aminopeptides (Fig. 4A). When the complemented *lapA* mutant was examined, we saw restoration of the six activities that were lacking in the mutant (Fig. 4A). The complement trended toward elevated levels of activity against alanine and serine aminopeptides (Fig. 4A), suggesting that increased amounts of LapA might cleave additional substrates.

To confirm the activities of LapA, we purified recombinant LapA-His6-tagged protein containing 378 amino acids of the mature *lapA* product (minus the 23-residue signal sequence). As expected, purified LapA had activity against leucine, valine, isoleucine, aspartate, methionine, and phenylalanine aminopeptides, with its activity being greatest against the leucine substrate (Fig. 4B). LapA lacked activity against proline, glutamate, and glycine, compatible with supernatant tests. Although assaying of supernatants had not revealed LapA-dependent cleavage of alanine, lysine, and serine aminopeptides, due to the presence of LapB and a T2S-independent factor (Fig. 4A) (30), the testing of purified enzyme affirmed LapA’s ability to cleave these substrates (Fig. 4B). Combining these data with past results (30), we conclude that LapA is active against ≥10 substrates. Most substrates had nonpolar, amino acid targets, but, given its cleavage of aspartate, lysine, serine, and tyrosine aminopeptides, LapA has rather broad activity (37, 38). Typically of a member of the M28 family (39), LapA was inhibited by bestatin (Fig. S4A).

10.1128/mBio.00528-18.4 FIG S4  Dose-dependent inhibition and substrate binding pocket of LapA. (A) Purified full-length LapA was incubated at 37°C with a leucine aminopeptidase substrate and 2-fold-increasing concentrations of bestatin. The residual aminopeptidase activity compared to that seen with untreated LapA was graphed against the log concentration of the inhibitor, and the half-maximal inhibitory concentration (IC_50_) was determined via nonlinear regression and sigmoidal slope response (variable slope) curve fitting (GraphPad Prism, version 7.00 for Windows; Graphpad Software, Inc., La Jolla, CA). Data are presented as mean residual activity with standard errors of results from two independent experiments (*n* = 2). (B) The S. griseus aminopeptidase/l-leucine complex (PDB code: 1F2O) and the A. proteolytica aminopeptidase/bestatin complex (PDB code: 1XRY) were superimposed on the aminopeptidase domain of LapA. The LapA substrate binding pocket is shown as light orange sticks, with modeled l-leucine and bestatin shown as green sticks. Download FIG S4, EPS file, 1.1 MB. Copyright © 2018 White et al. 2018 White et al. This content is distributed under the terms of the Creative Commons Attribution 4.0 International license.

LapA was predicted to contain an N-terminal, “protease-associated” (PA) domain, which in some M28 peptidases is necessarily excised in order to release a C-terminal peptidase domain (40, 41). That LapA might be processed is suggested from our prior two-dimensional (2-D) PAGE analysis of supernatants, which had detected truncated forms of LapA (28). To determine if processed LapA has activity, we purified a version of LapA that was missing the putative PA domain and repeated the enzyme assays. Truncated LapA had activity against the same substrates that had been cleaved by full-length LapA, with leucine aminopeptide remaining as the favored substrate (Fig. 4C). Interestingly, the activity against methionine aminopeptide and, to a lesser extent, the phenylalanine aminopeptide was elevated, whereas the activities against the other seven substrates were similar or slightly decreased (Fig. 4B and C). These data indicate that the removal of the PA domain of LapA, though not required, yields an enzymatically active protein. Also, full-length LapA and truncated LapA may have different substrate preferences.

**FIG 5:**
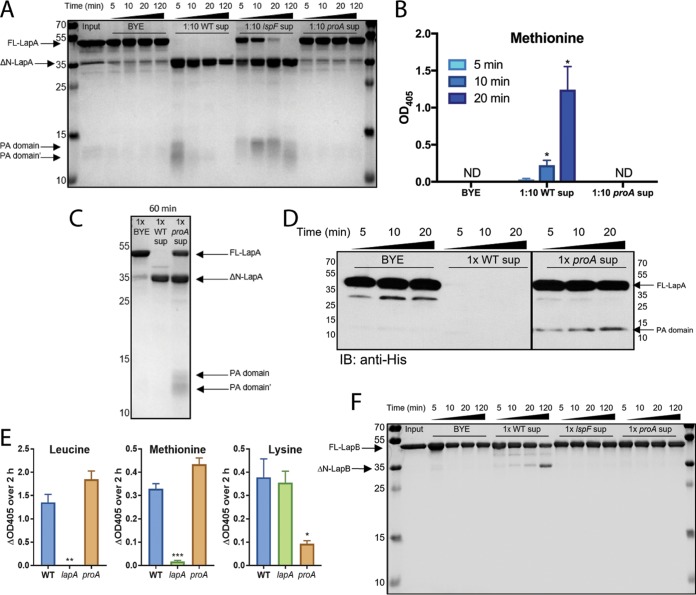
Effect of type II-secreted factors on LapA and LapB processing and activity. (A and B) Purified, full-length LapA (FL-LapA) (Input) was incubated at 37°C for the indicated times with either BYE medium or 1/10-diluted supernatants (sup) obtained from late-exponential-phase broth cultures of wild-type strain 130b (WT), *lspF* mutant NU275 (*lspF*), or *proA* mutant AA200 (*proA*) and then processed by SDS-PAGE followed by Coomassie staining for total protein detection (A) or incubated with methionine aminopeptidase substrate in order to assess LapA activation (B). Arrows to the left of the gel image in panel A denote the migration position of FL-LapA, LapA missing its N terminus (ΔN-LapA), the PA domain of LapA (PA domain), and smaller cleavage products (PA domain′). The identity of the minor bands that were present within the input material (including one that appears to nearly comigrate with ΔN-LapA) and were maintained during incubation with the BYE medium or culture supernatants is unknown, but they likely represented breakdown products of the recombinant LapA. The migration of molecular mass markers (in kilodaltons) is also denoted to the left of the gel in panel A. Enzyme activity data in panel B are presented as means with standard errors of results from three independent experiments (*n* = 3 each). Asterisks indicate significant increases in activity relative to *t* = 5 min (*, *P* < 0.05 [Student’s *t* test]). ND, not detected. (C and D) Purified FL-LapA was incubated at 37°C for the indicated times with either BYE medium or undiluted culture supernatants obtained from WT or *proA* mutant cultures and then processed for SDS-PAGE and Coomassie staining (C) or immunoblot analysis (IB) using anti-His antibodies directed against the hexahistidine tag embedded at the start of the PA domain (D). Arrows to the right side of the images in panels C and D denote the migration position of FL-LapA, ΔN-LapA, and the PA domain fragments, with the migration of the molecular mass markers denoted as well. Although the samples examined in panel D were on the same gel, they were not in adjacent lanes; therefore, we cropped out the intervening lanes that were not pertinent to the analysis. Data in panels C and D are representative of results of three and two experiments, respectively. (E) Filter-sterilized supernatants from late-exponential-phase broth cultures of wild-type 130b (WT), *lapA* mutant NU320 (*lapA*), and *proA* mutant AA200 (*proA*) were assayed for activity directed toward aminopeptidase substrates, with the N-terminal amino acid (AA) residue given. Changes in background-adjusted absorbance at OD_405_ for the AA–*p*-nitroanilide substrates were monitored kinetically over 2 h. Data are presented as means with standard errors of results from three independent experiments (*n* = 3 each). Asterisks indicate significant reduction in activity of a mutant relative to the WT (*, *P* < 0.05; **, *P* < 0.01; ***, *P* < 0.001 [Student’s *t* test]). (F) Purified, full-length LapB (FL-LapB) (Input) was incubated at 37°C for the indicated times with either BYE medium or undiluted supernatants obtained from late-exponential-phase broth cultures of wild-type strain 130b (WT), *lspF* mutant NU275 (*lspF*), or *proA* mutant AA200 (*proA*) and then processed by SDS-PAGE followed by Coomassie staining for total protein detection. Arrows to the left of the gel image denote the migration position of FL-LapB and of LapB missing its N terminus (ΔN-LapB). The migration of molecular mass markers (in kilodaltons) is also denoted to the left of the gel. Data are representative of results from 3 independent experiments.

We posited that the T2SS-dependent ProA protease might be cleaving secreted LapA into its N-terminal PA domain and C-terminal peptidase domain. ProA is the major secreted protein of L. pneumophila and is known to cleave other exoproteins (19, 35, 42, 43). Hence, we incubated recombinant LapA, with its His6 tag N terminal to its PA domain, along with supernatants (diluted 1/10) obtained from the wild-type 130b strain, the *lspF* mutant, or the *proA* mutant, and assayed for the cleavage of LapA by PAGE and Coomassie staining (Fig. 5A). Wild-type supernatants rapidly cleaved the ~44-kDa LapA, resulting in the appearance of an ~35-kDa C-terminal portion, an ~13-kDa PA domain, and smaller cleavage/degradation products. However, *proA* mutant supernatants exhibited no evidence of cleavage over 2 h of incubation, appearing to be equivalent in that respect to the BYE medium control (Fig. 5A). The *lspF* mutant supernatants were also deficient for activity, although cleavage products did appear, most likely due to some release of ProA as a result of cell lysis. Compatibly with these data, purified LapA treated with (1/10-diluted) wild-type supernatants, but not *proA* mutant supernatants or BYE medium, cleaved methionine aminopeptide (Fig. 5B). Thus, we infer that ProA cleaves and activates LapA. Interestingly, when purified LapA was mixed for 1 h with undiluted rather than diluted supernatants, ProA-independent cleavage of LapA was seen (Fig. 5C). When immunoblots were used to examine the effect of undiluted *proA* mutant supernatants, the low-level cleavage event (the appearance of His-tagged PA) was detected after 5 min (Fig. 5D). However, undiluted *proA* mutant supernatants retained activity against leucine and methionine aminopeptides (Fig. 5E). Incidentally, the *proA* mutant supernatants still lacked activity against lysine aminopeptide, a LapB substrate (Fig. 5E), and ProA-dependent cleavage of LapB was observed on stained gels (Fig. 5F). Overall, these data showed that ProA is mainly responsible for activation of LapA but that LapA can also be cleaved, albeit to a lesser extent, by another secreted factor.

### The structure of LapA.

**FIG 6:**
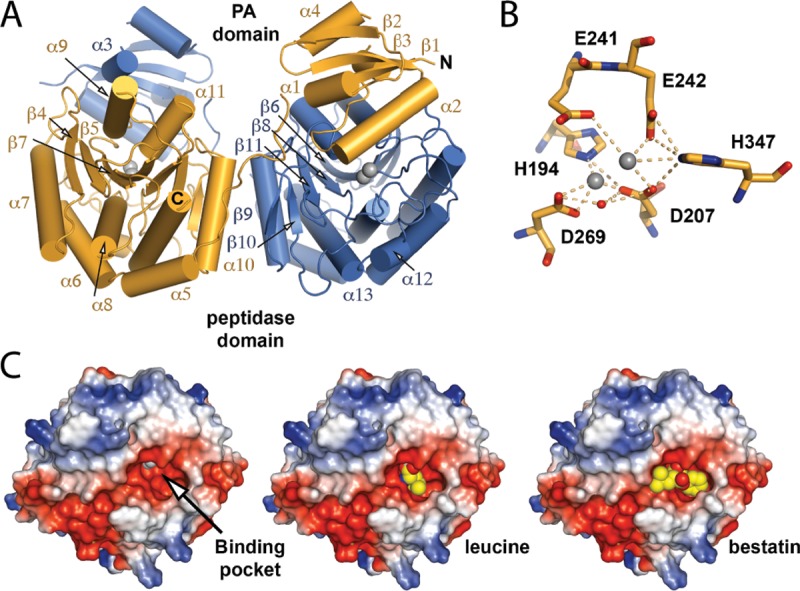
Overall structure of LapA. (A) The LapA domain-swapped dimer of the asymmetrical unit is presented as a cartoon with zinc ions drawn as gray spheres. (B) Active-site residues are shown as sticks, and zinc ions (gray) and water molecules (red) are drawn as spheres. (C) Electrostatic surface of the LapA aminopeptidase domain with active-site zinc ions as gray spheres (left). The electrostatic surface of LapA superimposed with (middle) the S. griseus aminopeptidase/l-leucine complex (PDB code: 1F2O) (48) or (right) the A. proteolytica aminopeptidase/bestatin complex (PDB code: 1XRY) (49), indicating potential binding of substrate amino acids, is shown. Leucine and bestatin are shown as spheres.

To determine how LapA provides its broad specificity, we began structural studies of recombinant LapA. Crystals were readily obtained, and its structure was determined by molecular replacement, using the coordinates of LapB (PDB code: 5GNE) (41) as the search model, and electron density maps were refined to 1.9 Å. The structure of LapA contains two molecules in the asymmetrical unit, and all residues could be built except for the N-terminal His tags and residues within the interdomain linkers (residues 82 to 101) that connect the PA domain (residues 1 to 81) and aminopeptidase domains (residues 102 to 378). The PA domain is formed of four helices and three β-strands, and the aminopeptidase domain has nine helices and seven β-strands. Although LapA was purified as a monomer, in our structure, the PA domain of one chain was associated with the aminopeptidase domain of an adjacent chain, which formed a domain-swapped dimer (Fig. 6A). The domain-swapped monomers were essentially identical, with a root mean square deviation (RMSD) over C_α_ atoms of 0.083 Å (without linkers), although variations were seen within the interdomain sequence. As an M28 family metalloprotease, the peptidase domain of LapA binds two Zn ions in its active site, which are coordinated by the conserved residues His194, Asp207, Glu241, Glu242, Asp269, and His347 and one water molecule (Fig. 6B). Drawing on structures of aminopeptidases from Streptomyces griseus (PDB code: 1F20) (44) and Aeromonas proteolytica (PDB code: 1XRY) (45), the main determinant of specificity in this class of enzyme is a binding pocket directly adjacent to the active site, and in LapA, this is formed from the side chains of Asp269, Met270, Phe285, Cys314, Cys318, Phe333, Cys335, Phe339, and His342 and from the backbone of Ser319, Thr337, and Ser338 (Fig. S4B). This creates a relatively deep hydrophobic cavity, which can readily accommodate aromatic and aliphatic residues (Fig. 6C; see also Fig. S4B). In our structure of LapA, the PA domain covers the active site and substrate-binding pocket (Fig. S5) and thus represents the proenzyme form of LapA. A ring of predominantly hydrophobic residues on the surface of the enzymatic domain (Trp170, Leu198, Asp199, Tyr316, Phe339, His342, His347) provides a platform for the PA domain to dock, and this is mediated through its β-1 strand (Glu7), α2-helix (Ser39, Glu43, Thr46, Asp50), α2-α3 linker (His56, Phe57, Asn59), and α3-helix (His62).

10.1128/mBio.00528-18.5 FIG S5  Interactions within the PA/aminopeptidase domain interface of LapA. (Left) The PA domain is shown as a cartoon, while the aminopeptidase domain is shown as a cartoon with a transparent surface. Interfacial residues are drawn as sticks. (Right) Magnification of the domain interface shown from three orientations. The secondary structure and the residues that make up the interface are annotated. Download FIG S5, EPS file, 3.1 MB. Copyright © 2018 White et al. 2018 White et al. This content is distributed under the terms of the Creative Commons Attribution 4.0 International license.

### LapA promotes nutrient acquisition during infection.

**FIG 7:**
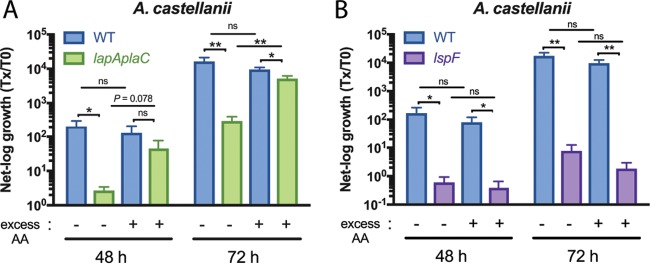
Intracellular infection of A. castellanii by the L. pneumophila wild-type strain, *lapA plaC* mutant, and *lspF* mutant in the presence of added amino acids. A. castellanii amoebas were infected with wild-type strain 130b (WT), *lapA plaC* mutant NU435, or *lspF* mutant NU275, and, at the indicated times, bacterial growth was determined. Where indicated, the medium in the infected wells was supplemented with 1 mM excess amino acids (AA) for the duration of the experiment. Growth data are presented as means with standard errors of results from three independent experiments (*n* = 3 each). Asterisks indicate significant differences in CFU recovery between strains under the given conditions (*, *P* < 0.05; **, *P* < 0.01 [Student’s *t* test]). ns, not significant.

Given the enzymatic activity of LapA, which was revealed by structural analysis, we posited that LapA promotes L. pneumophila growth in A. castellanii by cleaving aminopeptides to release amino acids for uptake by the parasite. Thus, we asked if addition of a cocktail of amino acids to the *Legionella*–*Acanthamoeba* coculture could rescue growth of the *lapA plaC* mutant. To facilitate this experiment, we employed an amino acid cocktail (aspartate, alanine, glutamate, asparagine, glycine, serine, and proline) that was previously used to rescue the growth of a L. pneumophila mutant that was starved due to its lack of a T4SS effector (46). Thus, we used a reagent that was not inhibitory to L. pneumophila or its host. Moreover, the aspartate, alanine, and serine in the cocktail are predicted products of LapA activity (Fig. 4). Whereas amino acid supplementation had no effect on wild-type growth, it increased *lapA plaC* mutant numbers at 72 h postinoculation, and the results trended toward increases at 48 h (Fig. 7A). Thus, amino acid supplementation phenocopied the complementation results (Fig. 3C). The supplementation did not rescue the *lspF* mutant (Fig. 7B), which is compatible with this mutant lacking many exoproteins. Since neither the wild type nor the T2SS mutant was affected by addition of amino acids, the rescue of the *lapA* mutant was not a nonspecific effect on A. castellanii or L. pneumophila. Thus, the LapA mutant’s infection defect is tied to its loss of enzyme activity. Given that LapA, PlaC, and amino acid acquisition does not fully account for the ≥1,000-fold defect observed upon complete loss of T2S (23), we sought additional secreted proteins that promote infection of amoebas.

### Proteomic and mutational analyses identified NttD as a novel T2SS-dependent exoprotein that also promotes infection of A. castellanii.

**FIG 8:**
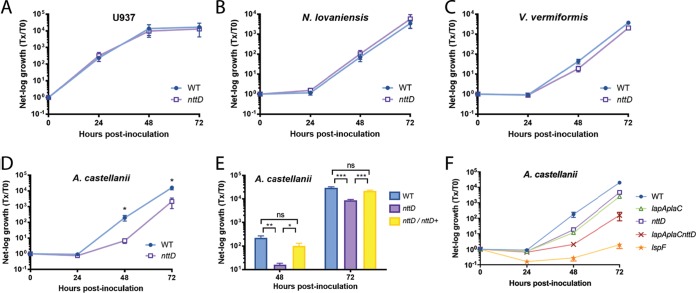
Intracellular infection of macrophages and amoebas by the L. pneumophila wild-type strain, an *nttD* mutant, a complemented *nttD* mutant, and a *lapA plaC nttD* mutant. (A to E) U937 cells (A), N. lovaniensis (B), V. vermiformis (C), and A. castellanii (D and E) were infected with wild-type strain 130b (WT), *nttD* mutant NU431, or complemented *nttD* mutant NU431 containing plasmid-carried *nttD*, and, at 0, 24, 48, and 72 h postinoculation, CFU levels in the infected wells were assayed. Bacterial growth resulting from intracellular infection is expressed as the ratio of the CFU level at *t* = 24, 48, or 72 h to the CFU level at *t* = 0. Data in panels A to D are presented as means with standard errors of results from at least three independent experiments (*n* ≥ 3). Data in panel E are presented as means with standard deviations of results from four infected wells and are representative of three independent experiments. Asterisks indicate significant differences in the levels of CFU recovery between the mutant and the WT or the complemented mutant (*, *P* < 0.05; **, *P* < 0.01; ***, *P* < 0.001 [Student’s *t* test]). (F) Amoebas were infected with the WT or *lapA plaC* mutant NU435, *nttD* mutant NU431, *lapA plaC nttD* mutant NU441, or *lspF* mutant NU275, and, at the indicated times, net bacterial growth was determined. Data are presented as means with standard errors of results from three independent experiments (*n* = 3 each). The *lapA plaC nttD* mutant displayed significantly reduced CFU recovery compared to the WT, the *lapA plaC* mutant, and the *nttD* mutant at 48 and 72 h postinoculation (*P* < 0.05 [Student’s *t* test]). The *lspF* mutant exhibited an even great reduction in growth compared to the WT, the *lapA plaC* mutant, and the *nttD* mutant at 24, 48, and 72 h (*P* < 0.01 [Student’s *t* test]).

We next studied a 41-kDa protein that was previously detected in supernatants of L. pneumophila wild-type strain 130b grown in BYE broth but was absent in similarly derived supernatants of an *lspF* T2S mutant (28). Inspection of the 130b genome (47) revealed that the T2SS-dependent protein is encoded by open reading frame (ORF) L. pneumophila lpw10421, the second gene in a four-gene operon (Fig. S2C). Given our proteomics data and the bioinformatic analysis that is to follow, we named lpw10421 *nttD*, for *n*ovel *t*ype *t*wo secreted protein D, following the nomenclature used to denote *nttA*, *nttB*, and *nttC* (23, 24). NttD is not annotated but is noted to have domain-of-unknown-function 4785. An unpublished crystal structure for NttD from *L. pneumophila* strain Philadelphia-1 has been deposited by others in the Protein Data Bank (PDB code: 4KH9). NttD is a mainly β-strand protein, and analysis using the PISA server (48) indicated that it exists as a dimer (Fig. S6A), although we detected equilibrium between the monomer and dimer in solution (Fig. S6B). Each chain is made of three unique domains, with the N-terminal domain mediating dimerization. While tertiary-structure analysis using the Dali server (49) detected no homologies for the N-terminal domain (no *Z* score of >8), the C-terminal domain shared features with glycosidases. Yet when we tested purified protein and concentrated supernatants against glycosidase substrates, no activity was seen. In order to discern a role for NttD, we made a mutant of 130b inactivated for *nttD* (NU431). The mutant grew in BYE broth like the parental 130b strain (Fig. S1A, right), indicating that NttD is not required for extracellular survival, at least under standard conditions. From examination of BYE supernatants, the mutant had normal levels of phosphatase, protease, and lipase activities (not shown), indicating that loss of NttD does not cause a general defect in T2S (24, 50). Whereas the intracellular replication of the *nttD* mutant was not affected in the human U937 cell macrophages and *Naegleria* and *Vermamoeba* amoebas (Fig. 8A to C), it was impaired for infection of A. castellanii (Fig. 8D). At 48 and 72 h postinoculation, the NU431-infected acanthamoebae yielded ~30-fold-fewer legionellae. When infections were done in a transwell apparatus, such that bacteria were prevented from contacting host cells, the wild type and the mutant, though not growing, survived similarly (Fig. S1B, right), indicating that the reduced recovery of the mutant from the coculture was a result of defective infection rather than of impaired survival in the medium. When we tested a second *nttD* mutant (NU432) for infection of A. castellanii, the same defect was seen (not shown), implying that the loss of infectivity was due to the mutation in *nttD* rather than to a second-site mutation. Because a wild-type level of growth was achieved upon reintroduction of *nttD* into mutant NU431 (Fig. 8E), we concluded that NttD is required for infection of A. castellanii. Together, these data document NttD as a novel, T2SS-dependent exoprotein that promotes L. pneumophila infection of A. castellanii, thereby enhancing the range of *Legionella* in aquatic habitats. Discovering substantial roles for NttD, PlaC, and LapA in L. pneumophila infection of A. castellanii, we pondered to what degree these proteins account for the role of T2SS in infection. Thus, we tested a new mutant (NU441) that lacks *lapA*, *plaC*, and *nttD*. The triple mutant had a sizeable defect that went from ~90-fold at 48 h postinoculation to ~130-fold at 72 h (Fig. 8F). This defect was greater than that of the *nttD* mutant and *lapA plaC* mutant (Fig. 8F), indicating that NttD and LapA/PlaC operate in distinct pathways. The *lapA plaC nttD* mutant’s defect did not fully recapitulate the *lspF* mutant’s defect (Fig. 8F), indicating that there are additional T2S-dependent proteins that contribute to intracellular infection. However, the NttD, LapA, and PlaC data do explain, to a significant degree, the role of T2S in infection of A. castellanii.

10.1128/mBio.00528-18.6 FIG S6  NttD forms monomers and dimers in solution. (A) The crystal structure of the NttD dimer (PDB code: 4KH9) reveals three domains per monomer, which are numbered 1 (purple), 2 (yellow), and 3 (red) from the N terminus to the C terminus and are given for chain A. Chain B is depicted entirely in green. The NttD structure appears to form a stable homodimer, which is mediated through N-terminal domain 1. (B) Gel filtration profile of NttD showing that it forms both monomers and dimers in solution. Download FIG S6, EPS file, 1.4 MB. Copyright © 2018 White et al. 2018 White et al. This content is distributed under the terms of the Creative Commons Attribution 4.0 International license.

### Bioinformatic analysis revealed distinctive distribution patterns for *nttD*-, *plaC*-, and *lapA-*related genes within and outside the *Legionella* genus.

**FIG 9:**
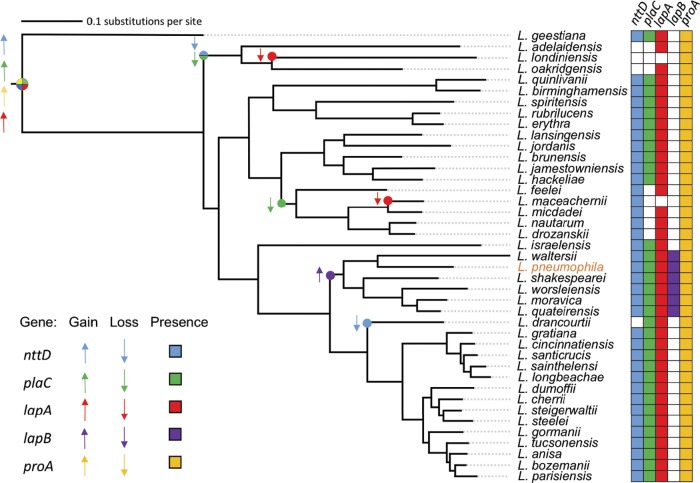
Distribution of genes encoding key type II-secreted proteins within the *Legionella* genus. Gain and loss events pertaining to genes encoding type II-secreted NttD, LapA, LapB, PlaC, and ProA were modeled on a maximum-likelihood tree of 41 *Legionella* species, which was constructed based on the protein sequence encoded by 78 nearly universal genes (9). Colored circles and arrows indicate a gene gain event (upward-pointing arrow) or loss event (downward-pointing arrow) based on maximum parsimony, and colored cells (right) indicate the presence of *nttD*, *lapA*, *lapB*, *plaC*, and *proA* within a given species.

All sequenced L. pneumophila strains and all of the 40 other sequenced species of *Legionella* have complete T2SSs (see Table S1 in the supplemental material). Therefore, we performed BLAST analyses to discern the species- and genus-wide distribution of *nttD*, *plaC*, and *lapA*. The *nttD* gene was in all L. pneumophila strains, and an *nttD*-like gene was in 36/40 *Legionella* species (Fig. 9; see also Table S2A). On the basis of the distribution of *nttD*-like genes across the phylogenetic tree, *nttD* was found to be an ancestral gene that, though largely conserved, has been lost twice (Fig. 9). Further BLAST analysis found that NttD shares relatedness to hypothetical proteins in ≥9 genera of gammaproteobacteria and in one genus of deltaproteobacteria (Table 1). Similarly to *nttD*, *plaC* was conserved in the L. pneumophila species (Table S2B). A *plaC*-like gene was evident in 32 other *Legionella* species (Fig. 9; see also Table S2B). The 8 remaining species lacked a *plaC*-like gene, indicating that the gene occurs in ~80% of species. We surmise that *plaC*, like *nttD*, is an ancestral gene that, though reasonably conserved, has been lost twice (Fig. 9). Like *nttD*, *plaC* was lost prior to emergence of the adelaidensis*-*londiniensis*-*oakridgensis clade. Yet *plaC* appears to have also been lost prior to the advent of the feeleii*-*micdadei-*drozanskii* clade, resulting in a lower level of conservation than was determined for NttD (Fig. 9). Looking beyond *Legionella*, we found homologues of PlaC in genera of gammaproteobacteria, although these genera were different from those that had NttD homologues (Table 2). Interestingly, most non-*Legionella* homologues of PlaC, including the closest homologues, were in *Cyanobacteria*, a distant phylum that does not have a canonical T2SS (Table 2) (13). BLAST revealed that *lapA* occurs in all L. pneumophila strains (Table S2C). *lapA*-like genes were in 38 of the other species of *Legionella* (Fig. 9; see also Table S2C). Only L. maceachernii and L. londiniensis did not carry a *lapA*-like gene, due to two “recent” gene loss events. Thus, *lapA* is in ~95% of species, indicating that it is an ancestral gene that is even more prevalent than *nttD* or *plaC* (Fig. 9). LapB, though conserved within the L. pneumophila species (Table S2D), is present in only 12% of non-pneumophila species (Fig. 9; see also Table S2D). Seemingly, *lapB* is a recent gene acquisition, coincident with emergence of the clade containing L. pneumophila (Fig. 9). We also wondered about the prevalence of ProA, the T2SS-dependent protease that activates LapA and PlaC (Fig. 5) (35), and found that *proA* was 100% conserved (Fig. 9). Returning to the distribution of LapA-like proteins but looking beyond *Legionella*, BLAST analysis found that LapA’s closest prokaryotic homologues occur in betaproteobacteria (Table 3). This pattern of distribution of non-*Legionella* homologues was very different from that of NttD and PlaC, which included primarily gammaproteobacteria or cyanobacteria and no betaproteobacteria (Tables 1 and 2). Even more interestingly, the closest relative, which had 41% identity and 60% similarity with LapA, was an aminopeptidase of A. castellanii (Table 3). Other eukaryotic LapA-like proteins were in fungi, and in many cases, they were closer to LapA than the prokaryotic homologues (Table 3). Thus, LapA has strong eukaryotic-protein-like traits.

10.1128/mBio.00528-18.8 TABLE S1  Conservation of the T2SS apparatus genes. This table lists the T2SS apparatus genes present within L. pneumophila strains (A) and among different *Legionella* species (B). Download TABLE S1, PDF file, 0.03 MB. Copyright © 2018 White et al. 2018 White et al. This content is distributed under the terms of the Creative Commons Attribution 4.0 International license.

10.1128/mBio.00528-18.9 TABLE S2  Distribution of the *nttD*, *plaC*, *lapA*, and *lapB* genes. This table lists the *nttD* (A), *plaC* (B), *lapA* (C), and *lapB* (D) genes present within L. pneumophila strains and among different *Legionella* species. Download TABLE S2, PDF file, 0.1 MB. Copyright © 2018 White et al. 2018 White et al. This content is distributed under the terms of the Creative Commons Attribution 4.0 International license.

**TABLE 1:** BLAST results for NttD outside the *Legionella* genus^a^

Organism^b^	I, S, C^c^	E value	Accession no.	Classification
Dyella japonica	25, 45, 89	1E−22	WP_035323882.1	Gammaproteobacteria
Oleiagrimonas soli	25, 44, 80	2E−21	WP_043100684.1	Gammaproteobacteria
Nannocystis exedens	23, 43, 80	1E−19	WP_096328563.1	Deltaproteobacteria
Arenimonas oryziterrae	24, 41, 85	3E−18	WP_022968800.1	Gammaproteobacteria
Kangiella aquimarina	26, 44, 72	2E−16	WP_018624440.1	Gammaproteobacteria
Lysobacter capsici	23, 40, 86	7E−13	WP_082723375.1	Gammaproteobacteria
Microbulbifer donghaiensis	24, 46, 80	2E−12	WP_073272160.1	Gammaproteobacteria
Teredinibacter turnerae	25, 45, 80	1E−11	WP_018414733.1	Gammaproteobacteria
Rhodanobacter spathiphylli	22, 41, 83	1E−11	WP_007808810.1	Gammaproteobacteria
Stenotrophomonas maltophilia	22, 38, 78	4E−10	WP_042612098.1	Gammaproteobacteria

a Analysis of non-*Legionella* homologues was done with the exclusion of hits from the *Legionella* genus only with homologues predicted using an E-value cutoff of <4 × 10^−10^ and an alignment length of at least 65% (80).

b Only the top representative species per genus is given, based on the closest homology with NttD.

c I, percent identity; S, percent similarity; C, percent coverage.

**TABLE 2:** BLAST results for PlaC outside the *Legionella* genus

Organism^a^	I, S, C^b^	E value	Accession no.	Classification
*Anabaena cylindrical*	28, 47, 69	5E−32	WP_015214616.1	Cyanobacteria
Cylindrospermum stagnale	27, 44, 70	4E−29	WP_015210343.1	Cyanobacteria
Endozoicomonas arenosclerae	29, 43, 95	3E−27	WP_062262828.1	Gammaproteobacteria
Anabaenopsis circularis	23, 41, 72	1E−26	WP_096581537.1	Cyanobacteria
Fischerella muscicola	26, 42, 68	6E−26	WP_051035591.1	Cyanobacteria
Tolypothrix bouteillei	25, 43, 69	7E−26	WP_038073042.1	Cyanobacteria
Chlorogloeopsis fritschii	27, 42, 71	9E−26	WP_016872501.1	Cyanobacteria
Mastigocladopsis repens	28, 43, 69	1E−25	WP_033366504.1	Cyanobacteria
Scytonema millei	25, 42, 75	3E−25	WP_069349443.1	Cyanobacteria
Hassallia byssoidea	26, 42, 68	2E−24	KIF33255.1	Cyanobacteria

a Only the top representative species per genus is given, based on the closest homology with PlaC. After the genera listed here, the next seven encoding a PlaC-like protein (E value, <1E−18) are five cyanobacteria followed by two gammaproteobacteria.

b I, percent identity; S, percent similarity; C, percent coverage.

**TABLE 3:** BLAST results of LapA outside the *Legionella* genus

Organism^a^	I, S, C^b^	E value	Accession no.	Classification
Acanthamoeba castellanii	41, 60, 70	7E−65	XP_004341372.1	Eukarya—protozoa
Chromobacterium sphagni	40, 59, 70	2E−64	WP_071111733.1	Betaproteobacteria
Chitinimonas koreensis	40, 59, 70	5E−64	WP_028447211.1	Betaproteobacteria
Burkholderia thailandensis	39, 59, 69	5E−61	WP_019254803.1	Betaproteobacteria
Pseudogulbenkiania ferrooxidans	38, 58, 70	8E−61	WP_021478877.1	Betaproteobacteria
Massilia yuzhufengensis	39, 57, 70	2E−60	WP_091877370.1	Betaproteobacteria
Pleurotus ostreatus	37, 53, 75	8E−60	KDQ28511.1	Eukarya—fungi
Duganella sacchari	33, 53, 92	9E−60	WP_072788963.1	Betaproteobacteria
Janthinobacterium agaricidamnosum	37, 57, 72	2E−59	WP_038494963.1	Betaproteobacteria
Syncephalastrum racemosum	39, 58, 68	5E−59	ORY97700.1	Eukarya—fungi
Coprinopsis cinerea	37, 57, 71	7E−59	XP_001831818.1	Eukarya—fungi
Actinomucor elegans	36, 58, 68	9E−59	AME15509.1	Eukarya—fungi
Rhizophagus irregularis	36, 57, 69	2E−58	ESA22882.1	Eukarya—fungi
Trichosporon asahii	37, 56, 72	7E−58	EKD01610.1	Eukarya—fungi
Roseateles aquatilis	38, 56, 70	1E−57	WP_088383330.1	Betaproteobacteria
Conidiobolus coronatus	38, 57, 69	1E−57	KXN70597.1	Eukarya—fungi
Pseudoduganella violaceinigra	34, 55, 81	3E−57	WP_028103074.1	Betaproteobacteria
Cylindrobasidium torrendii	34, 53, 77	3E−57	KIY62743.1	Eukarya—fungi
Ulvibacter litoralis	40, 55, 69	3E−57	WP_093139368.1	Bacteroidetes
Mucor circinelloides	36, 58, 69	5E−57	EPB89639.1	Eukarya—fungi
Schizophyllum commune	36, 55, 69	8E−57	XP_003037075.1	Eukarya—fungi
Pseudoalteromonas byunsanensis	36, 56, 79	1E−56	WP_070992923.1	Gammaproteobacteria

a Only the top representative species per genus is given, based on the closest homology with LapA. After the genera listed here, the next 20 encoding a LapA-like protein (E value, <1E−53) are 15 genera of fungi and 1 genus each of betaproteobacteria, gammaproteobacteria, deltaproteobacteria, *Actinobacteria*, and *Bacteroidetes*.

b I, percent identity; S, percent similarity; C, percent coverage.

## DISCUSSION

The experimental data presented increase our understanding of T2S and host-parasite interactions in multiple ways. With the definition of NttD and LapA as major potentiators of infection of A. castellanii, the number of T2S effectors confirmed as being involved in infection of amoebas rises to seven (23, 24, 30, 31). Thus, approximately one-third of the known effectors are required for infection (13), implying that intracellular parasitism has played a large role in shaping the T2SS repertoire. That NttD, along with the previously described NttA and NttC, has a role in amoebal infection and yet lacks similarity to known enzymes suggests that many T2SS substrates may be specialized for the intra-amoebal lifestyle (23, 24). Although aminopeptidases in the M28 family have been linked to T2S and/or virulence (37, 51, 52), the results from this study of LapA mark the first documentation of a secreted aminopeptidase promoting an intracellular infection event. Thus, prokaryotic LapA-like proteins (Table 3) may be facilitating survival for bacteria such as *Duganella* and *Burkholderia* spp. that reside in amoebas (53). In establishing a link between LapA and PlaC, we have also revealed a unique example of functional redundancy operating during infection. While promoting growth in A. castellanii, NttD and the combination of LapA and PlaC were not needed for infection of other amoebas. This highlights how the importance of T2SS substrates varies in a host-specific fashion; presumably, each amoebal type represents a distinct environment and, hence, L. pneumophila invokes different exoproteins in order to efficiently establish its host range. Unlike NttD and LapA, PlaC was critical for infection of every amoebal host. There are probably commonalties across different amoebas, and so L. pneumophila repeatedly relies upon some of its effectors to flourish in aquatic habitats. The finding that ProA processes LapA and LapB adds to past work showing that ProA cleaves PlaA and PlaC and is required for strain 130b infection of V. vermiformis and N. lovaniensis (30, 35, 43). Another study found that a *proA* (*mspA*) mutant of strain JR-32 is impaired for infection of A. castellanii (19). Thus, ProA rivals PlaC in its broad significance. Although previous studies reported on the transcription of a few T2S genes (54–56), the current analysis represents the largest assessment of T2S effector expression by L. pneumophila. After examining 19 genes in two stages of extracellular growth and in three hosts, it is apparent that secreted-protein genes exhibit a variety of expression patterns rather than showing similar responses to conditions. From this, we intuit that the amounts of secreted protein made are controlled at the level of the individual or of subsets of effector-gene transcription more than at the level of apparatus gene transcription.

**FIG 10:**
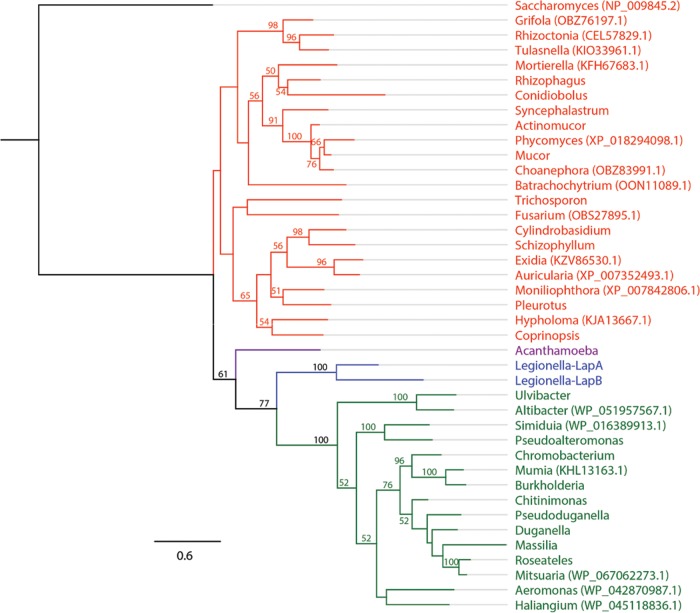
Interkingdom phylogenetic tree of LapA-like proteins. A maximum-likelihood tree shows 41 aligned amino acid sequences of M28 family aminopeptidases. Bootstrap values (from 250 replicates) of >50 are given at the corresponding nodes. Aminopeptidase Y from Saccharomyces cerevisiae was used as the outgroup. The scale bar indicates the number of amino acid substitutions per site. Fungi are indicated in red, protozoa in purple, and bacteria in green, with the exception of L. pneumophila proteins, which are indicated in blue. NCBI accession numbers not listed in Table 3 are noted in parentheses after the respective genus designations.

Bioinformatic analyses revealed further insights into T2SS and the origins of its effectors. Being found in the majority of *Legionella* species, the genes encoding NttD, PlaC, and LapA appear to be ancestral genes that underwent only a few loss events during the evolution of the genus. On the basis of the dispersal of homologues in other types of organisms, ancestral NttD was likely acquired from related gammaproteobacterial species, PlaC from cyanobacterial species, and LapA from protozoa or fungi. ProA is encoded by another ancestral T2SS gene, but in this case, no loss events were detected. Unlike NttD, PlaC, LapA, and ProA, LapB was only in the “recent” clade containing L. pneumophila. We posit that LapB arose from *lapA* duplication. This is supported by phylogenetic analysis of LapA and LapB, which form a monophyletic group exclusive of other bacteria or eukaryotes, and is based on the fact that LapB has acquired substitutions relative to the ancestral LapA (Fig. 10). Given that NttD, PlaC, LapA, and ProA promote infection but LapB does not, we speculate that ancestral, highly conserved T2SS effectors have the greater role in intracellular parasitism. This evolutionary pattern for T2SS effectors differs from that of most T4SS effectors, which are distributed in <25% of *Legionella* species (9).

The activities associated with PlaC and LapA provide hypotheses for how these T2S substrates promote infection. PlaC not only generates fatty acids but also can modify lipids within amoebal vacuoles (34, 35). Thus, PlaC may promote intracellular nutrient acquisition via direct lipid metabolism, or through modification of lipids in LCV membranes, which might in turn influence vesicular trafficking or anchoring of other effectors. LapA likely promotes replication through its ability to generate amino acids that are limiting during the intracellular lifecycle. Our analysis found a broad spectrum of activity for LapA, and we showed that amino acid supplementation rescues the growth of mutants lacking LapA. Interestingly, the leucine, methionine, isoleucine, and valine generated by LapA, coupled with arginine produced by LapB (30), account for all of the amino acids that cannot be synthesized by L. pneumophila or A. castellanii (46). It is intriguing to contemplate why LapA and PlaC, two very different exoenzymes, should overlap in terms of function. In one scenario, L. pneumophila utilizes both amino acids (generated by LapA) and lipids (generated by PlaC) for nutrition within the LCV. Thus, a growth defect is revealed only when both LapA and PlaC are absent, depriving the bacterium of two food sources. Alternatively, changes to LCV membranes mediated by PlaC might promote the import of nutrients beyond those targeted by LapA. Hence, without PlaC and LapA, intravacuolar bacteria again lose two food sources. To discern how PlaC and LapA operate, it would be helpful to learn their locations in the infected cell. Proteins secreted by T2S, including ProA, can be translocated out of the LCV (57), and thus, it is possible that LapA or PlaC or both have targets in the host cytoplasm. Overall, LapA and PlaC modulate different processes and yet accomplish a single task.

With the characterization of LapA, we have a striking example of a eukaryotic-protein-like, T2SS-dependent exoprotein, indicating that eukaryotic-protein-like effectors of L. pneumophila are not limited to the T4SS (58). LapA was likely acquired through horizontal gene transfer within A. castellanii or an ancestral protozoan. Indeed, the top-scoring LapA homologue outside *Legionella* belongs to A. castellanii and is a putative aminopeptidase. Also, LapA shares higher-than-average homology to its top-scoring eukaryotic homologue (41% identity, 60% similarity), compared to the average levels of identity and similarity of all other (primarily T4SS) eukaryotic-protein-like proteins of L. pneumophila (34% identity, 52% similarity) (59). Moreover, phylogenetic reconstruction of aligned amino acid sequences of LapA homologues revealed that LapA is not descended from other bacteria but shares a common ancestor with A. castellanii (Fig. 10). Interestingly, the A. castellanii aminopeptidase is secreted (60). Thus, legionellae benefit from secreting a host-like aminopeptidase as a means of scavenging limiting amino acids.

The structure of LapA is similar to those of several known aminopeptidases. The Dali server identified LapB (41) and A. proteolytica Apap (PDB code: 1AMP) (61) as having the highest tertiary homology (*Z* scores of 54.6 Å and 45.0 Å, respectively), although this is concentrated at the peptidase domain (see Fig. S7A in the supplemental material). For example, the positions of active-site residues in LapA and LapB structures are almost identical (Fig. S7B), while there is a significant reorganization of helices between PA domains. This accommodates differences in their substrate-binding pockets, with LapB’s cavity being wider and more negatively charged (Fig. S7C), compatible with a preference for positively charged lysine and arginine (30). Often, the role of the PA domain is to keep an enzyme in an inactive state prior to secretion (62), although for purified LapA, it did not prevent detection of activity. The effect of the PA domain is likely due to a transient association between the PA and peptidase domains in LapA, as we observed a monomeric and domain-swapped form in solution. Although domain swapping was not seen in the structure of LapB, it dimerizes in solution and its PA domain does not prevent detection of activity under some conditions (41). The LapA structure is the fifth structure for a *Legionella* T2SS effector, joining those of NttD, LapB, phosphatase Map, and VirK-like Lpg1832 (28, 41, 63–65).

10.1128/mBio.00528-18.7 FIG S7  Comparison of the L. pneumophila LapA and LapB tertiary structure, active-site residues, and substrate binding pocket. (A) Significant deviations between the LapA (orange) and LapB (PDB code: 5GNE) (purple) structures were observed within the PA domain helices, which are annotated. (B) Amino acid residues of the active sites of LapA (orange) and LapB (purple) are drawn as sticks, and zinc (Zn) and water (WAT) molecules are drawn as spheres. (C) LapA and LapB structures are shown as electrostatic surface potential data with active-site zinc ions drawn as gray spheres. Substrate binding sites are highlighted with a yellow dashed line. Download FIG S7, EPS file, 4.4 MB. Copyright © 2018 White et al. 2018 White et al. This content is distributed under the terms of the Creative Commons Attribution 4.0 International license.

Although the PlaC, LapA, and NttD data explain, to a large extent, the impact of T2S on L. pneumophila infection of A. castellanii, future work should further define the *Legionella* T2SS output. Proteomic assessment of supernatants and subsequent (single) mutant analysis have heretofore been the means to identifying T2SS-dependent proteins that promote infection (13). These approaches helped reveal the nature of NttD as a potentiator of infection and will continue to offer new targets for analysis (28). Yet this strategy clearly failed to reveal the roles that LapA and PlaC have in A. castellanii infection (23, 30), undoubtedly due to the redundancy that exists between LapA and PlaC. The new-found importance of these two effectors was uncovered through a combination of transcriptional and mutational analyses. This approach has four steps: (i) identification of the genes that are upregulated during wild-type infection of the host; (ii) examination of the transcript profile of mutants that lack the upregulated genes, while being alert to compensatory increases in other transcripts; (iii) mutation of two or more upregulated genes that exhibit a compensatory link; and (iv) testing of mutants for loss of infection. Our data set predicts more substrates that may promote infection; e.g., two strongly upregulated genes during infection of A. castellanii and N. lovaniensis were *legP* encoding a putative protease and *map* encoding a phosphatase (Fig. 1B and C). Though neither a *legP* mutant nor a *map* mutant was impaired for infection (23), a *legP map* mutant might be defective. Interestingly, LegP and Map, like LapA, have eukaryotic-protein-like homologs (23, 28, 64).

## MATERIALS AND METHODS

### Strains, plasmids, and media.

L. pneumophila 130b (ATCC BAA-74) served as the wild type and parent for all mutants (25). *proA* mutant AA200, *lspF* mutant NU275, *lapA* mutant NU320, *lapB* mutant NU322, *lapA lapB* mutant NU324, *celA* mutant NU353, and *plaC* mutants NU367 and NU420 were described before (14, 15, 23, 29, 30, 66). Newly derived mutants lacking *nttD*, *lapA*, or *plaC* were made using methods of allelic exchange (23, 25). Complemented mutants were obtained by introducing intact copies of *nttD*, *lapA*, or *plaC* on a plasmid, analogously to past studies (24). Primers and plasmids used in mutant and complement constructions are listed in Table S3 in the supplemental material. Legionellae were routinely grown at 37°C on buffered charcoal yeast extract (BCYE) agar or in BYE broth (25). Escherichia coli DH5α (Life Technologies) was the host for the recombinant plasmids used in mutant construction and complementation. E. coli K-12 SHuffle (NEB) was used to get purified protein. E. coli cells were grown in LB medium. Unless otherwise noted, chemicals were from Sigma.

10.1128/mBio.00528-18.10 TABLE S3  Primers and plasmids. This table lists the primers (A) and plasmids (B) that were used in this study. Download TABLE S3, PDF file, 0.1 MB. Copyright © 2018 White et al. 2018 White et al. This content is distributed under the terms of the Creative Commons Attribution 4.0 International license.

### Infection assays.

U937 cells (ATCC CRL-1593.2), A. castellanii (ATCC 30234), V. vermiformis (ATCC 50237), and N. lovaniensis (ATCC 30569) were infected with L. pneumophila as previously described (23). To assess survival of L. pneumophila in A. castellanii-conditioned media, we inoculated legionellae and amoebas on opposite sides of a 0.4-µm-pore-size transwell membrane (Corning; catalog no. 3470). To that end, 1 × 10^5^ amoebas in 1 ml of protease-peptone-yeast extract (PY) medium (23) were seeded into 24-well plates, and then 1 × 10^4^ wild-type or mutant bacteria were added in 100 µl PY medium to the transwell support. After periods of incubation, a 10-µl aliquot from the upper chamber was plated on BCYE agar to determine numbers of legionellae. To test the effect of amino acid supplementation on bacterial growth in A. castellanii, an amino acid cocktail (Lonza; catalog no. 13-114E) was added to the PY medium, giving 1 mM of each amino acid.

### Quantitative reverse transcriptase PCR.

qRT-PCR was done as previously described (17). To monitor L. pneumophila transcription during extracellular growth, RNA was isolated from BYE cultures using a GeneJET RNA purification kit (Thermo Scientific). To examine bacterial RNA from infected host cells, U937 cells, A. castellanii, and N. lovaniensis were infected as described above, with the exception that the inoculation step was facilitated by centrifugation (500 × *g*, 5 min) of bacteria onto the monolayer, and the infected cells were lysed and processed using the GeneJET kit. Primers RW12 to RW69 (Table S3) used for qRT-PCR were designed using the Primer-BLAST tool at NCBI. lpw00031 (*gyrB*) and lpw16991 were used as references to normalize gene expression (67), and changes in expression were calculated using the threshold cycle (2^−ΔΔ*CT*^) method (68).

### Protein purifications.

Full-length LapA (FL-LapA; residues 1 to 378), N-terminal PA domain-truncated LapA (ΔN-LapA; residues 89 to 378), full-length LapB (FL-LapB; residues 1 to 373), and full-length NttD (FL-NttD; residues 1 to 373), minus N-terminal signal sequences, were amplified from 130b DNA and cloned into N-terminal His-tagged pET-46 Ek/LIC (EMD Millipore), using primers JG1 to JG8 (Table S3). Expression of the recombinant proteins in E. coli was induced with 1 mM IPTG (opyl-β-d-thiogalactopyranoside) at an optical density at 600 nm (OD_600_) of 0.6, and cells were harvested after overnight growth at 18°C with shaking. Cells were suspended in a mixture containing 20 mM Tris-HCl (pH 8), 200 mM NaCl, 5 mM MgCl_2_, 1 mg/ml DNase I, and 5 mg/ml lysozyme and were lysed by sonication. Recombinant proteins were purified using nickel-affinity chromatography (Qiagen) followed by gel filtration using a Superdex 75 column (GE Healthcare) equilibrated in 20 mM Tris–HCl (pH 8)–200 mM NaCl.

### Enzyme assays.

Cell-free supernatants collected from late-log BYE cultures were assayed for protease, acid phosphatase, and lipase activities as previously described (50, 64). To assess secreted aminopeptidase activity, two previously described methods were used (30). Leucine, lysine, methionine, glycine, aspartate, isoleucine, proline, glutamate, and valine aminopeptidase activities were measured by the release of *p*-nitroanilide (*p*-NA) from l-leucine *p*-NA, l-lysine *p*-NA, l-methionine *p*-NA, glycine *p*-NA, l-aspartate *p*-NA (Chem-Impex), l-isoleucine *p*-NA (Chem-Impex), l-proline *p*-NA (MP Biomedicals), l-glutamate *p*-NA (Chem-Impex), and l-valine *p*-NA (Chem-Impex), respectively (30). Phenylalanine, alanine, and serine aminopeptidase activities were measured by release of β-naphthylamide (β-NA) from l-phenylalanine β-NA, l-alanine β-NA, and l-serine β-NA (Chem-Impex), respectively (30). Purified LapA-FL and LapA-ΔN were diluted to a concentration of 500 nM in the reaction buffers and assayed as described above. One unit of activity is equal to that which catalyzes the conversion of 1 µmol substrate/min, and molecular activity was expressed as the number of units per micromole of purified enzyme, based on standard curves corresponding to *p*-NA and β-NA (30, 69). Assays for α-mannosidase, β-mannosidase, β-galactosidase, and endo-1,4-β-mannanase were as previously described (70–72).

### Assays for the cleavage, activation, and inhibition of aminopeptidases.

To assay for the ability of secreted proteins to cleave aminopeptidases, 10 µg of purified LapB-FL or LapA-FL was incubated at 37°C for up to 2 h with 50 µl of 1× or 1:10-diluted late-log-phase culture supernatants, respectively. Samples (10 µl) were collected on ice and were then subjected to SDS-PAGE. Protein was visualized with SimplyBlue SafeStain (Thermo Scientific). Immunoblots were done as previously described (17), with recombinant protein detected with mouse anti-His (Millipore; clone HIS.H8). For real-time monitoring of LapA-FL cleavage and resultant activation, LapA-FL (500 nM) was added to the assay buffer containing 3 mM *p-*NA-based substrate and the supernatant (1:10 final concentration). Absorbance was monitored at 405 nm. LapA inhibition assays were done using 500 nM LapA-ΔN incubated with serial dilutions of bestatin. Residual activity following treatment was determined based on release of *p-*NA from l-leucine–*p-*NA as described above.

### Crystal structure determination.

Crystallization of LapA (10 mg/ml) was done using the sitting-drop vapor-diffusion method and growth at 293 K in 0.15 M ammonium sulfate–0.1 M 4-morpholineethanesulfonate (pH 5.5)–25% (wt/vol) polyethylene glycol 4000. Crystals were soaked for 30 to 60 s in cryoprotection solution (well solution supplemented with 20% glycerol) and flash-cooled in liquid nitrogen. Diffraction data were collected at 100 K on Beamline I03 at the Diamond Light Source (DLS), United Kingdom. Data were processed using XDS (73) and scaled using AIMLESS (74). Initial phases were obtained using PHASER (75) with the structure of LapB (41) as the search model. Density modification was done with PARROT (76) followed by automated model building with ARPWARP (77). The remaining structure was manually built within COOT (78), and refinement was carried out with refmac (79) using noncrystallographic symmetry and translation-libration-screw groups. Ten percent of the reflections were omitted for cross-validation. Processing and refinement statistics for the final model are given in Table 4.

**TABLE 4:** Data collection and refinement statistics

Parameter	LapA result(s)^a^
Crystal parameters	
Space group	*P*2_1_2_1_2_1_
Cell dimensions (Å)	*a* = 76.60; *b* = 99.95; *c* = 104.02
	
Data collection	
Beamline	DLS I03
Wavelength (Å)	0.97625
Resolution (Å)	99.55–1.87 (1.87–1.92)
No. of unique observations	66,414 (4,808)
*R*_sym_^b^	0.063 (1.2)
<*I*>/*σI*	12.5 (3.7)
Completeness (%)	100 (100)
Redundancy	6.5 (6.2)
	
Refinement^c^	
* R*_work_/*R*_free_ (%)	19.2/23.8
No. of protein residues	719
No. of water molecules	313
No. of ions	4 Zn ions
	
RMSD stereochemistry^d^	
Bond lengths (Å)	0.022
Bond angles (°)	2.045
	
Ramachandran analysis^e^	
Residues in outlier regions (%)	0.0
Residues in favored regions (%)	98.6
Residues in allowed regions (%)	100

a Numbers in parentheses refer to the outermost resolution shell.

b *R*_sym_ = ∑|*I* − <*I*>|/∑*I*, where *I* is the integrated intensity of a given reflection and <*I*> is the mean intensity of multiple corresponding symmetry-related reflections.

c *R*_work_ = ∑||F_o_ − |F_c_||/∑F_o_, where F_o_ and F_c_ are the observed and calculated structure factors, respectively. *R*_free_ = *R*_work_ values calculated using 10% random data excluded from the refinement.

d The RMSD stereochemistry value represents the deviation from ideal values.

e Ramachandran analysis was carried out using Molprobity (81).

### Accession number(s).

Coordinates and structure factors have been deposited in the wwPDB under PDB code 6ESL.
