# Supplementary material for: Type II Secretion-Dependent Aminopeptidase LapA and Acyltransferase PlaC Are Redundant for Nutrient Acquisition during Legionella pneumophila Intracellular Infection of Amoebas
Source: mBio. 2018 Apr 17;9(2):e00528-18. doi: 10.1128/mBio.00528-18 (PMC5904407; doi:10.1128/mBio.00528-18)
Supplement: TABLE S1 [file mbo002183833st1.pdf]

Table S1A: Conservation of T2SS apparatus genes among *L. pneumophila* strains.

		Percent identity to the *lsp* gene of *L. pneumophila* strain 130b											
Strain	Source	*lspC*	*lspD*	*lspE*	*lspF*	*lspG*	*lspH*	*lspI*	*lspJ*	*lspK*	*lspL*	*lspM*	*lspO/pilD*
130b	clinical	100	100	100	100	100	100	100	100	100	100	100	100
Paris	clinical	99.41	99.37	98.79	98.50	100	94.57	97.6	99.02	97.20	97.63	97.44	98.95
Lens	clinical	100	99.49	98.79	99.25	100	98.91	99.2	100	99.07	99.21	99.36	99.65
Philadelphia-1	clinical	99.41	99.12	98.99	98.25	100	98.37	99.2	99.51	98.45	98.94	98.72	99.65
Sudbury	clinical	100	99.12	98.99	98.25	100	98.37	99.2	99.51	98.45	98.94	98.72	99.65
Thunderbay	clinical	100	99.12	98.99	98.25	100	98.21	99.2	99.51	98.45	98.94	98.72	99.65
Detroit-1	clinical	100	97.35	96.36	96.49	98.57	91.30	92.8	93.66	92.55	89.71	94.87	91.29
ATCC 43290	clinical	100	99.12	98.99	98.25	100	98.37	99.2	99.51	98.45	98.94	98.72	99.65
LPE509	environmental	99.41	99.12	98.99	98.25	100	98.37	99.2	99.51	98.45	98.94	98.72	99.65
Lorraine	clinical	100	99.49	98.79	98.75	100	95.11	97.6	99.02	98.76	98.42	98.72	99.30
Mississauga	clinical	98.82	98.74	98.58	98.50	100	96.20	96	99.02	98.45	97.89	98.08	96.52
Toronto-2005	clinical	100	99.37	98.99	98.50	100	95.11	97.6	99.02	97.83	98.42	97.44	98.61
Corby	clinical	99.41	98.74	98.58	98.50	100	96.20	96	99.02	98.14	97.89	98.08	98.61
ATCC 33215	clinical	99.41	99.12	98.99	98.25	100	98.37	99.2	99.51	98.45	98.94	98.72	99.65
Alcoy	clinical	100	98.74	98.58	98.50	100	96.20	96	99.02	98.14	97.89	98.08	98.61
ATCC 43283	clinical	100	98.74	98.58	98.75	100	93.48	97.6	99.02	98.14	97.89	98.08	96.52
ATCC 33737	environmental	99.41	96.21	96.76	96.24	99.29	89.13	92.8	94.63	94.41	89.45	96.15	88.85

Table S1B: Conservation of T2SS apparatus genes within the *Legionella* genus.[a]

			Percent identity to the *lsp* gene of *L. pneumophila* strain 130b										
Species	Strain	Source	*lspC*	*lspD*	*lspE*	*lspF*	*lspG*	*lspH*	*lspI*	*lspJ*	*lspK*	*lspL*	*lspM*	*lspO/pilD*
pneumophila	130b	clinical	100	100	100	100	100	100	100	100	100	100	100	100
adelaidensis	ATCC49625	environmental	65.00	63.06	77.32	71.68	83.94	45.40	59.65	51.52	50.33	38.88	44.08	61.38
anisa	WA-316-C3	environmental	63.91	75.00	88.69	82.71	94.20	61.01	73.50	70.65	58.17	61.38	63.06	68.29
birminghamensis	CDC#1407-AL-14	clinical	41.29	67.82	79.39	76.94	87.59	46.25	60.33	58.91	49.02	40.89	43.51	58.04
bozemanii	WIGA	clinical	61.31	76.10	89.07	83.21	94.93	59.12	73.50	70.71	57.00	61.38	61.78	68.64
brunensis	ATCC43878	environmental	46.11	66.91	83.20	77.69	85.40	51.68	72.95	68.47	52.94	44.19	47.10	60.74
cherrii	ORW	environmental	61.18	75.57	89.70	81.45	94.93	61.40	70.69	69.85	57.10	61.48	61.15	72.13
cincinnatiensis	CDC#72-OH-14	clinical	56.81	75.47	87.88	81.70	93.48	61.54	68.55	71.14	59.48	58.05	63.46	69.69
drancourtii	LLAP12	environmental	62.72	74.30	89.29	83.58	92.75	65.69	71.67	partial[b]	70.94	59.37	66.67	67.60
drozanskii	ATCC700990	environmental	60.78	64.66	83.00	79.20	86.86	45.46	72.41	68.14	52.27	48.18	45.16	65.13
dumoffii	NY-23	environmental	60.36	75.28	89.29	80.95	94.93	62.58	73.50	69.19	57.98	60.42	61.78	72.13
erythra	SE-32A-C8	environmental	47.65	71.00	78.50	77.19	89.05	46.15	64.80	55.45	47.71	40.84	44.87	63.64
feeleii	WO-44C	environmental	60.95	70.44	81.74	80.15	84.67	45.75	72.17	58.91	51.47	43.72	45.16	61.19
geestiana	ATCC49504	environmental	54.29	65.47	77.89	71.68	82.86	44.67	54.39	52.53	40.00	34.65	37.82	48.23
gomanii	LS-13	environmental	60.00	75.28	89.50	82.71	93.48	58.62	66.67	71.14	60.59	61.91	59.62	71.78
gratiana	ATCC49413	environmental	58.58	74.72	88.69	82.46	92.75	62.13	68.55	72.86	59.67	57.52	64.74	67.73
hackeliae	798-PA-H	clinical	44.05	66.88	82.59	77.19	84.67	48.41	73.91	67.98	55.12	44.07	47.68	56.79
israelensis	Bercovier 4	environmental	58.58	68.48	81.71	74.62	81.30	53.79	67.50	65.84	48.69	46.31	44.87	64.69
jamestowniensis	JA-26-G1-E2	environmental	59.05	66.29	83.43	77.89	85.40	50.97	71.05	62.56	51.79	42.56	44.81	59.72
jordanis	BL-540	environmental	57.14	66.42	82.79	75.94	83.94	47.74	67.83	61.77	50.65	42.34	41.45	61.13
lansingensis	ATCC49751	clinical	47.13	66.22	83.43	78.95	83.21	46.15	68.64	68.63	53.75	46.39	48.68	59.09
londiniensis	ATCC49505	environmental	57.43	66.20	79.75	72.11	78.83	51.15	60.87	53.03	48.20	40.42	39.10	61.62
longbeachae	NSW150	clinical	56.00	74.56	88.08	81.96	93.48	63.87	69.60	71.14	58.82	59.47	64.10	68.64
maceachernii	PX-1-G2-E2	environmental	47.62	65.38	83.60	77.69	88.32	48.05	69.83	60.10	52.60	45.23	44.74	62.64
micdadei	TATLOCK	clinical	61.77	67.18	84.41	79.45	86.86	46.75	74.36	62.56	52.27	45.95	42.76	64.18
moravica	ATCC43877	environmental	68.64	81.46	89.68	85.43	93.53	70.24	80.83	75.39	64.84	62.53	73.72	73.52
nautarum	ATCC49506	environmental	61.77	64.63	83.00	79.70	89.78	46.58	72.41	68.14	50.65	46.35	45.81	66.90
oakridgensis	Oak Ridge-10	environmental	60.58	67.13	81.41	77.14	83.57	50.85	66.96	53.96	50.33	43.75	49.03	64.79
parisiensis	PF-209-C-C2	environmental	61.31	76.07	89.50	82.96	94.93	60.38	73.50	71.72	57.00	60.32	60.51	69.34
quateirensis	ATCC49507	environmental	61.31	76.07	89.50	82.96	94.93	60.38	73.50	71.72	57.00	60.32	60.51	69.34
quinlivanii	CDC#1442-AUS-E	environmental	41.03	68.58	81.82	76.44	86.86	45.75	61.02	58.50	47.71	40.31	38.96	58.54
rubrilucens	WA-270A-C2	environmental	46.47	70.04	79.11	77.44	88.32	45.63	65.08	55.67	48.38	40.31	44.23	64.36
sainthelensi	Mt.St.Helens-4	environmental	59.17	75.06	88.28	82.46	92.03	59.76	69.36	71.14	60.13	59.37	63.46	70.38
santicrucis	SC-63-C7	environmental	57.40	74.94	88.49	82.71	92.75	61.54	68.29	70.65	57.52	60.00	63.46	71.08
shakespearei	ATCC49655	environmental	69.41	84.20	87.85	86.50	93.53	70.91	79.31	77.83	66.88	60.69	67.31	71.78
spiritensis	Mt.St.Helens-9	environmental	42.86	69.32	81.58	78.64	86.13	47.21	67.77	56.65	53.42	44.13	45.46	62.55
steelei	IMVS3376	clinical	59.76	75.82	88.46	81.96	93.48	63.52	70.94	70.85	59.28	59.52	65.61	71.43
steigerwaltii	SC-18-C9	environmental	61.18	76.20	89.68	81.70	92.75	56.22	70.16	70.20	58.69	60.58	64.97	72.13
tucsonensis	ATCC49180	clinical	63.31	76.13	89.09	83.46	92.75	61.78	73.28	67.16	57.66	61.38	62.42	71.08
waltersii	ATCC51914	environmental	63.91	82.24	87.25	85.18	97.14	63.10	74.14	80.10	66.99	59.00	68.59	67.02
worsleiensis	ATCC49508	environmental	67.46	79.95	88.06	85.71	92.09	69.64	78.13	73.40	60.34	57.41	67.95	69.34
Avg. identity			57.38	71.91	85.14	79.99	89.56	55.20	69.79	66.06	55.33	51.54	54.25	65.71

[a] Homologues within the *Legionella* genus were conservatively predicted using an E value cutoff of $<1x10^{-20}$ and an alignment length of at least 80% (Gomez-Valero L, Rusniok C, Rolando M, Neou M, Dervins-Ravault D, Demirtas J, et al. Genome Biol 15:505, 2014).

[b] the *lspJ* gene of *L. drancourtii* lies at the end of a contig, thus we cannot conclude whether the entire gene is intact.
