# Supplementary material for: Type II Secretion-Dependent Aminopeptidase LapA and Acyltransferase PlaC Are Redundant for Nutrient Acquisition during Legionella pneumophila Intracellular Infection of Amoebas
Source: mBio. 2018 Apr 17;9(2):e00528-18. doi: 10.1128/mBio.00528-18 (PMC5904407; doi:10.1128/mBio.00528-18)
Supplement: TABLE S2 [file mbo002183833st2.pdf]

Table S2A: Presence of *nttD* within *L. pneumophila* and throughout the genus.

L. pneumophila **strain**[a]	**% identity**	**% similarity**	**% coverage**	**E value**
130b[b]	100	100	100	0
Paris[b]	100	100	100	0
Lens[b]	100	100	100	0
Philadelphia-1[b]	99.74	100	100	0
Sudbury	99.74	100	100	0
Thunderbay	99.74	100	100	0
Detroit-1	99.74	100	100	0
ATCC 43290	99.74	100	100	0
LPE509	99.74	100	100	0
Lorraine	99.49	100	100	0
Mississauga	98.47	99.23	100	0
Toronto-2005	98.47	99.23	100	0
Corby	98.47	99.23	100	0
ATCC 33215	98.47	99.23	100	0
Alcoy	98.21	99.23	100	0
ATCC 43283	98.21	99.23	100	0
ATCC 33737	95.41	97.45	100	0

[a] see Table S1 for source designation.

[b] ORF lpw10421 (*nttD*) of strain 130b corresponds to lpg0956 of strain Philadelphia-1, to lpp1018 of strain Paris, and to lpl0985 of strain Lens.

Legionella **species**[c]	**% identity**	**% similarity**	**% coverage**	**E value**
pneumophila[d]	95-100	97-100	100	0
quateirensis	70.66	85.97	100	0
moravica	69.90	85.46	100	0
shakespearei	69.90	84.69	100	0
waltersii	69.64	84.95	100	0
worsleiensis	64.89	81.42	100	0
anisa	53.42	72.41	100	3.24E-150
bozemanii	53.17	71.90	100	5.66E-150
parisiensis	53.17	71.39	100	1.66E-147
gormanii	52.78	72.22	100	7.17E-151
tucsonensis	52.66	71.39	100	2.05E-147
steigerwaltii	51.65	71.90	100	4.60E-149
cherrii	51.39	69.37	100	2.98E-141
santicrucis	51.27	72.08	100	8.10E-148
gratiana	51.27	72.08	100	8.45E-148
cincinnatiensis	51.02	71.57	100	3.19E-146
longbeachae	50.76	71.07	100	1.55E-144
sainthelensi	50.40	71.77	96.68	1.46E-140
steelei	49.87	70.38	100	1.71E-140
dumoffii	48.35	68.35	100	3.13E-135
israelensis	46.57	67.43	100	6.42E-128
lansingensis	42.03	60.76	100	2.38E-105
rubrilucens	42.03	60.00	100	4.71E-103
hackeliae	41.88	61.93	100	6.07E-107

jamestowniensis	41.41	60.86	100	1.73E-105
feeleii	41.03	60.26	99.49	9.98E-104
spiritensis	40.51	59.49	99.49	9.68E-100
erythra	40.36	59.14	100	5.39E-99
brunensis	39.95	60.81	100	1.39E-99
nautarum	38.69	60.22	93.62	2.34E-83
drozanskii	38.48	60.21	97.45	4.48E-90
jordanis	38.33	57.11	100	5.38E-89
maceachernii	37.63	61.36	100	1.99E-91
birminghamensis	36.64	57.76	100	1.02E-86
quinlivanii	36.61	56.23	96.17	6.86E-79
micdadei	36.04	58.63	100	9.78E-90
geestiana	31.08	50.63	100	3.32E-61
adelaidensis		no gene detected		
drancourtii		no gene detected		
londiniensis		no gene detected		
oakridgensis		no gene detected		

[c] see Table S1 for strain and source designation.

[d] based on the 17 representative pneumophila strains appearing at the top of Table S2.

Table S2B: Presence of *plaC* within *L. pneumophila* and throughout the genus.

L. pneumophila **strain**	**% identity**	**% similarity**	**% coverage**	**E value**
130b[e]	100	100	100	0
Lens[e]	100	100	100	0
Toronto-2005	99.77	100	100	0
Paris[e]	99.54	100	100	0
Philadelphia-1[e]	99.54	100	100	0
LPE509	99.54	100	100	0
Lorraine	99.54	100	100	0
Mississauga	99.54	100	100	0
Alcoy	99.54	100	100	0
ATCC 43283	99.54	100	100	0
Sudbury	99.54	100	100	0
Thunderbay	99.54	100	100	0
ATCC 43290	99.54	100	100	0
ATCC 33215	99.54	100	100	0
Corby	99.31	100	100	0
Detroit-1	98.15	99.54	100	0
ATCC 33737	97.92	99.77	100	0

[e] ORF lpw30971 (*plaC*) of strain 130b corresponds to lpg2837 of strain Philadelphia-1, to lpp2894 of strain Paris, and to lpl2749 of strain Lens.

Legionella **species**	**% identity**	**% similarity**	**% coverage**	**E value**
pneumophila	97-100	99-100	100	0
waltersii	77.55	90.05	99.8	0
quateirensis	72.98	85.45	100	0

moravica	72.69	85.19	99.8	0
cincinnatiensis	72.24	85.88	98.2	0
shakespearei	72.22	85.65	99.8	0
santicrucis	72.00	85.88	98.2	0
longbeachae	72.00	85.88	98.2	0
sainthelensi	72.00	85.65	98.2	0
bozemanii	71.16	83.72	99.3	0
tucsonensis	71.16	83.95	99.3	0
steelei	71.16	84.19	99.3	0
cherrii	71.06	84.94	98.2	0
anisa	70.82	83.76	98.2	0
gormanii	70.82	84.00	98.2	0
steigerwaltii	70.82	85.18	98.2	0
gratiana	70.82	85.41	98.2	0
worsleiensis	70.56	85.28	98.8	0
parisiensis	70.47	83.95	99.3	0
dumoffii	70.47	84.19	99.3	0
drancourtii	70.23	85.58	99.3	0
hackeliae	67.37	80.75	98.4	0
brunensis	67.29	81.88	98.2	0
lansingensis	66.59	82.12	98.2	0
jordanis	65.41	80.71	98.2	0
jamestowniensis	65.26	79.58	98.4	0
spiritensis	64.37	79.81	97.2	0
erythra	63.36	78.49	97.7	0
rubrilucens	62.76	77.75	98.6	0
birminghamensis	62.56	76.98	99.3	0
quinlivanii	62.13	78.29	100	0
israelensis	62.12	77.88	98.2	0
geestiana	53.77	69.58	97.9	4.46E-171
adelaidensis		no gene detected		
drozanskii		no gene detected		
feelei		no gene detected		
londiniensis		no gene detected		
maceachernii		no gene detected		
micdadei		no gene detected		
nautarum		no gene detected		
oakridgensis		no gene detected		

Table S2C: Presence of *lapA* within *L. pneumophila* and throughout the genus.

***L. pneumophila* strain**	**% identity**	**% similarity**	**% coverage**	**E value**
130b[f]	100	100	100	0
Toronto-2005	99.75	100	100	0
Paris[f]	99.75	100	100	0
Philadelphia-1[f]	99.75	100	100	0
LPE509	99.75	100	100	0

Lorraine	99.75	100	100	0
Sudbury	99.75	100	100	0
Thunderbay	99.75	100	100	0
ATCC 43290	99.75	100	100	0
ATCC 33215	99.75	100	100	0
Alcoy	99.50	99.50	100	0
ATCC 43283	99.50	99.50	100	0
Corby	99.50	99.50	100	0
Lens[f]	97.01	99.50	100	0
Mississauga	97.01	99.50	100	0
Detroit-1	96.26	99.00	100	0
ATCC 33737	96.26	98.50	100	0

[f] ORF lpw30701 (*lapA*) of strain 130b corresponds to lpg2814 of strain Philadelphia-1, to lpp2866 of strain Paris, and to lpl2729 of strain Lens.

Legionella **species**	**% identity**	**% similarity**	**% coverage**	**E value**
pneumophila	96-100	98-100	100	0
quateirensis	79.30	88.53	95.02	0
shakespearei	79.05	88.78	95.02	0
moravica	78.55	89.03	95.02	0
worsleiensis	76.06	86.78	95.02	0
drancourtii	75.56	85.54	95.02	0
waltersii	75.31	87.03	95.02	0
gormanii	71.89	83.83	95.26	0
cherrii	69.90	82.84	95.26	0
bozemanii	69.65	82.84	95.26	0
tucsonensis	69.65	83.33	95.26	0
longbeachae	69.58	82.79	95.02	0
parisiensis	69.40	82.84	95.26	0
anisa	69.15	82.84	95.26	0
santicrucis	69.08	83.79	95.02	0
steigerwaltii	68.97	82.84	96.21	0
steelei	68.91	83.33	95.26	0
cincinnatiensis	68.47	81.53	96.21	0
gratiana	68.08	82.04	95.02	0
dumoffii	67.91	82.09	95.26	0
sainthelensi	67.83	83.54	95.02	0
israelensis	67.00	81.50	94.79	0
spiritensis	66.75	81.64	95.50	0
erythra	66.33	79.80	95.02	0
brunensis	65.67	78.61	95.26	0
rubrilucens	65.59	79.05	95.02	0
lansingensis	65.34	80.30	95.02	0
jordanis	63.34	78.30	95.02	0
hackeliae	63.09	78.05	95.02	0
drozanskii	62.40	76.21	92.65	0
jamestowniensis	62.34	77.06	95.02	0
birminghamensis	61.69	74.88	95.26	0
quinlivanii	60.95	75.62	95.26	0

micdadei	60.85	75.56	95.02	0
nautarum	60.35	75.31	95.02	0
feeleii	60.10	77.31	95.02	0
geestiana	57.75	77.25	94.79	3.54E-178
oakridgensis	56.75	73.75	94.79	2.85E-169
adelaidensis	54.78	71.83	91.71	6.47E-152
londiniensis		no gene detected		
maceachernii		no gene detected		

Table S2D: Presence of *lapB* within *L. pneumophila* and throughout the genus.

***L. pneumophila* strain**	**% identity**	**% similarity**	**% coverage**	**E value**
130b[g]	100	100	100	0
Lens[g]	98.74	99.24	100	0
Toronto-2005	99.24	99.50	100	0
Paris[g]	97.73	98.49	100	0
Philadelphia-1[g]	97.48	98.74	100	0
LPE509	97.73	98.49	100	0
Lorraine	97.23	98.49	100	0
Mississauga	98.24	98.99	100	0
Alcoy	98.24	98.99	100	0
ATCC 43283	98.24	98.99	100	0
Sudbury	97.48	98.74	100	0
Thunderbay	97.48	98.74	100	0
ATCC 43290	97.48	98.74	100	0
ATCC 33215	97.48	98.74	100	0
Corby	98.24	98.99	100	0
Detroit-1	89.92	95.21	100	0
ATCC 33737	89.92	94.96	100	0

[g] ORF lpw00321 (*lapB*) of strain 130b corresponds to lpg0032 of strain Philadelphia-1, to lpp0031 of strain Paris,and to lpl0032 of strain Lens.

***Legionella* species**	**% identity**	**% similarity**	**% coverage**	**E value**
pneumophila	89-100	94-100	100	0
quateirensis	63.98	78.34	100	0
moravica	63.57	76.63	100	0
shakespearei	62.92	78.77	98.49	0
waltersii	61.21	76.83	100	1.19E-180
worsleiensis	60.20	76.83	100	0
adelaidensis		no gene detected		
anisa		no gene detected		
birminghamensis		no gene detected		
bozemanii		no gene detected		
brunensis		no gene detected		
cherrii		no gene detected		
cincinnatiensis		no gene detected		
drancourtii		no gene detected		

drozanskii	no gene detected
dumoffii	no gene detected
erythra	no gene detected
feeleii	no gene detected
geestiana	no gene detected
gormanii	no gene detected
gratiana	no gene detected
hackeliae	no gene detected
israelensis	no gene detected
jamestowniensis	no gene detected
jordanis	no gene detected
lansingensis	no gene detected
londiniensis	no gene detected
longbeachae	no gene detected
maceachernii	no gene detected
micdadei	no gene detected
nautarum	no gene detected
oakridgensis	no gene detected
parisiensis	no gene detected
quinlivanii	no gene detected
rubrilucens	no gene detected
sainthelensi	no gene detected
santicrucis	no gene detected
spiritensis	no gene detected
steelei	no gene detected
steigerwaltii	no gene detected
tucsonensis	no gene detected
