# Supplementary material for: Type II Secretion-Dependent Aminopeptidase LapA and Acyltransferase PlaC Are Redundant for Nutrient Acquisition during Legionella pneumophila Intracellular Infection of Amoebas
Source: mBio. 2018 Apr 17;9(2):e00528-18. doi: 10.1128/mBio.00528-18 (PMC5904407; doi:10.1128/mBio.00528-18)
Supplement: TABLE S3 [file mbo002183833st3.pdf]

Table S3A: Primers used in this study

Primer	Description	Sequence (5' to 3')*
RW1	*nttD* 5' F	ATGGTGCCAACATCATGCGT
RW2	*nttD* 5' R	AAAAAACCCGGGTAATGCGAACGACAGCGCCA
RW3	*nttD* 3' F	AAAAAACCCGGGAATCAGGATGACGAGGTTGGCA
RW4	*nttD* 3' R	TAGCCAACGCTATGCCCTCAAA
RW5	*nttD* compl F	AAAAAAGGTACCAAAATGAAAACTGCCCACCTT
RW6	*nttD* compl R	AAAAAATCTAGATGCCCTCAAACAAAGTGACA
RW7	*lapA* compl F	AAAAGGTACCTGTAGTATATTTGCTCATTGATG
RW8	*lapA* compl R	AAAAAAGCTTATTTATGAGAGGCTTTAAAACAG
RW9	*plaC* compl F	AAAAGAATTCTGCATTTATTGGATATCGGGATT
RW10	*plaC* compl R	AAAAGAATTCCCACTGTTAATGTGGTTCAGGTG
RW11	*plaC* scr R	ACCGCTTCTGCGTTCTGATT
RW12	*lspF* qPCR F	TTCTATTTGCAGCGGGAGTC
RW13	*lspF* qPCR R	GCCTCACTGATTCCACTTCC
RW14	*lipA* qPCR F	CCTTGCACGGTTGGTTAGAT
RW15	*lipA* qPCR R	TCCTGGGGGTAAGTGTGAAG
RW16	*lipB* qPCR F	TGCAAGGACAAACAGAGCAG
RW17	*lipB* qPCR R	AGCCAATACTAGCCGCTGAC
RW18	*plaA* qPCR F	GCGCATGTGATGGTTATTTG
RW19	*plaA* qPCR R	ACTCCACACCAGCTTCATCC
RW20	*srnA* qPCR F	CGCTCATCGTGGTGATACTG
RW21	*srnA* qPCR R	CACCAAGATCCCATTCTTGC
RW22	*celA* qPCR F	CAGCACGGCAGTAGAGTTCA
RW23	*celA* qPCR R	ATTACCGGCTTCAGGTTGTG
RW24	*lapB* qPCR F	CCCTCCGTCGTCACTGTAAT
RW25	*lapB* qPCR R	TAGAGTCATCATCGGCACCA
RW26	*lapA* qPCR F	GGTTACCGTGATTGGCAAAG
RW27	*lapA* qPCR R	ATGCTCGATGATCCACTTCC
RW28	*chiA* qPCR F	CTGCATTTTCCCAGCTATCC
RW29	*chiA* qPCR R	TTAGCCGTTCCCTCGATATG
RW30	*legP* qPCR F	ATGAGCAATCCCGATCAGAC
RW31	*legP* qPCR R	TCACCGAAATCTTTCCCATC
RW32	*plaC* qPCR F	TTGTCTCTCCCTTTGGTTGG
RW33	*plaC* qPCR R	AAAACGCAGGATCCTCTTCC
RW34	*plcA* qPCR F	AACAGTTTTGCCAGGAATGC
RW35	*plcA* qPCR R	CTTTTGCAATCCCTGATTCG
RW36	*plcB* qPCR F	GGTCATCTTAGACGCCATGC

RW37	*plcB* qPCR R	AGTCGGGAAACACATCCATC
RW38	*lspD* qPCR F	GCCGCGAGCTATATCAAGTC
RW39	*lspD* qPCR R	TCATATCGTCACCTCGTGGA
RW40	*lspE* qPCR F	AGCTATCGCTCCTTTGGTCA
RW41	*lspE* qPCR R	CCACTCGTTCTCCATGGTTT
RW42	*map* qPCR F	ATGAGCGCCCAGTCATTGTT
RW43	*map* qPCR R	AGACGGTGCGCTAAATACGG
RW44	*proA* qPCR F	ACTCCCGACACTCAATCGAC
RW45	*proA* qPCR R	AACCTGCATACAATGCGTCA
RW46	*csrA* qPCR F	AGGCAATCAAGTTCGCTTAGG
RW47	*csrA* qPCR R	CTGCTTGTTCCGAATCATCAGA
RW48	*lspG* qPCR F	GGATGAAGCCCGTAAAGTCA
RW49	*lspG* qPCR R	GGAACAGGATTGCTTGTTGG
RW50	*lspC* qPCR F	ATCTGCCAGTGGGGAAGAA
RW51	*lspC* qPCR R	AAGGCTTAGCGACAGGTTCA
RW52	*lspL* qPCR F	ACTGGTTTGCGCTAGCATCT
RW53	*lspL* qPCR R	AGGCGACAGGTATTTTGCTG
RW54	*gamA* qPCR F	CGTTATCCCGGCGATACTTA
RW55	*gamA* qPCR R	TTCGGGGACAGATTATGAGC
RW56	LPW16991 qPCR F	GATTCCGGCCTAAAGGAAAC
RW57	LPW16991 qPCR R	GCTGCACCCTTGCTTTTAAC
RW58	*gyrB* qPCR F	AATCCCACTGCAGCAAAATC
RW59	*qyrB* qPCR R	TGGTAAACCGGCAATATCCA
RW60	*flaA* qPCR F	GGTTGGGGCGAATTCAAACC
RW61	*flaA* qPCR R	GTTGCTGCTCCTCCTCCAAT
RW62	*nttB* qPCR F	TGCGTGAACAGCTGTCAGAA
RW63	*nttB* qPCR R	TGGCGTTAACTGCATTCCCA
RW64	*nttC* qPCR F	GCCTACATAGCAGGCAGCAT
RW65	*nttC* qPCR R	GCAGGGCATTGACCATTAGT
RW66	*nttA* qPCR F	TCCAATGCTGAGGACACAGC
RW67	*nttA* qPCR R	GCATGCTGGCATACAGTTCA
RW68	*nttD* qPCR F	AATGATCGCGGCGAAAACTG
RW69	*nttD* qPCR R	CTTACCGGGTTCAAGCCACT
JG1	FL-LapA F	GACGACGACAAGATGACGACGTCGCCTGTTCATG
JG2	FL-LapA R	GAGGAGAAGCCCGGTTATTACTCGGAAGCGAGTTCAATAGC
JG3	dN-LapA F	GACGACGACAAGATGGGTGTATCCAAATTACATAAAGACGTTTATG
JG4	dN-LapA R	GAGGAGAAGCCCGGTTATTACTCGGAAGCGAGTTCAATAGC
JG5	FL-LapB F	GACGACGACAAGATGCAGTCCCCTGAAGAAGATAGAATC

JG6	FL-LapB R	GAGGAGAAGCCCGGTTACTAATTTAAACCAAGCTCTACAATAAAGG
JG7	FL-NttD F	GACGACGACAAGATGTTTACATTACCAAGACAACCAACCAAATC
JG8	FL-NttD R	GAGGAGAAGCCCGGTTAATCCACTAACTGACTAAGCTTGAC

* Restriction sites are underlined

Table S3B: Plasmids used in this study

Plasmid	Description	Reference
pGEM-T Easy	mutagenizing vector backbone (Amp^R)	Promega
pGnttDa	pGEM containing 5' *nttD* (Amp^R)	this study
pGnttDb	pGEM containing 3' *nttD* (Amp^R)	this study
pX1918-GT	Gentamicin cassette donor vector	Tyson JY et al. Infect Immun. 2013;81:1399-410
pGnttD::Gt	*nttD* mutagenizing plasmid (Gent^R)	this study
pGplaC::Kn	*plaC* mutagenizing plasmid (Kan^R)	McCoy-Simandle K et al. Infect Immun. 2011;79:1984-97
pGplaC::Cm	*plaC* mutagenizing plasmid (Chloro^R)	Tyson JY et al. Infect Immun. 2013;81:1399-410
pMMB2002	complementation vector backbone (Chloro^R)	Rossier et al. Infect Immun. 2004;72:310-21
pMnttD	*nttD* complementation plasmid (Chloro^R)	this study
pMMB-Gent	complementation vector backbone (Gent^R)	Hammer BK and Swanson MS. Mol Microbiol. 1999;33:721-31
pMlapA	*lapA* complementation plasmid (Gent^R)	this study
pMplaC	*plaC* complementation plasmid (Gent^R)	this study
